# Single-Atom Cu Anchored on a UiO-66 Surface-Enhanced Raman Scattering Sensor for Trace and Rapid Detection of Volatile Organic Compounds

**DOI:** 10.34133/research.0841

**Published:** 2025-08-21

**Authors:** Yuening Wang, Xiangyu Meng, Wenxiong Shi, Yujiao Xie, Aochi Liu, Lei Xu, Lin Qiu, Xiaoyu Song, Mingjian Zhang, Jiahao Zhang, Jian Yu, Aiguo Wu, Xiaotian Wang, Jie Lin

**Affiliations:** ^1^ School of Chemistry, Beihang University, Beijing 100191, China.; ^2^ School of Basic Medical Sciences, Hebei University, Baoding 071002, China.; ^3^ State Key Laboratory of Separation Membranes and Membrane Processes, School of Materials Science and Engineering, Tiangong University, Tianjin 300387, China.; ^4^ Laboratory of Advanced Theranostic Materials and Technology, Ningbo Institute of Materials Technology and Engineering, Chinese Academy of Sciences, Ningbo 315201, China.; ^5^ School of Energy and Environmental Engineering, University of Science and Technology Beijing, Beijing 100083, China.; ^6^ Key Laboratory of Jiangxi Province for Persistent Pollutants Control and Resources Recycle, Nanchang Hangkong University, Nanchang 330063, China.

## Abstract

Volatile organic compounds (VOCs) serve as critical biomarkers in exhaled breath for early-stage cancer patients, and their rapid, trace-level detection holds marked implications for cancer screening. Surface-enhanced Raman scattering (SERS) technology demonstrates strong potential for trace VOC gas detection due to its ultra-high sensitivity and immunity to water interference. However, while surface plasmon resonance (SPR)-free semiconductor substrates offer superior spectral stability and selectivity, their sensitivity toward VOC detection remains suboptimal. This study introduces a novel semiconductor-based SERS substrate composed of copper single atoms anchored on UiO-66 (Cu_1_/UiO-66), achieving a record-low detection limit of 10 parts per billion for VOC gases with a rapid 2-min response time, thereby elevating the gas-sensing performance of SPR-free substrates to unprecedented levels. The exceptional SERS activity originates from the highly delocalized electron properties of single-atomic copper, which effectively facilitates single-atom charge transfer processes. Concurrently, the incorporation of copper single atoms modulates the band structure of UiO-66, substantially enhancing the coupling resonance between the substrate and target molecules. In simulated breath tests mimicking lung cancer patients’ exhalations, Cu_1_/UiO-66 exhibits remarkable VOC recognition capability and robust anti-interference performance. This work pioneers a new paradigm for ultra-sensitive, rapid detection of trace VOCs in exhaled breath, holding substantial promise for early cancer diagnostics and clinical translation.

## Introduction

The exhaled breath of cancer patients contains specific volatile organic compounds (VOCs), and the analysis of their species and concentrations can be used for early diagnosis of cancer [1,2]. Various aldehydes/ketones (such as acetaldehyde and acetone) and various aromatic compounds (such as toluene, benzaldehyde, and furan) have been identified as major gaseous biomarkers for lung cancer or gastric cancer [3–5]. At present, multiple detection methods have been developed for VOC detection, such as fluorescence spectroscopy, magnetic resonance imaging, and gas chromatography–mass spectrometry [6–9]. Nevertheless, these techniques are heavily dependent on costly equipment or intricate manipulation techniques, resulting in numerous challenges for their applications. Therefore, there is a need to develop a rapid and sensitive detection method for VOCs. Surface-enhanced Raman scattering (SERS), recognized as an ultra-sensitive, non-destructive, and real-time fingerprint spectroscopy technique, has been widely applied in various fields [10–15]. However, the rapid diffusion kinetics of gas pose a challenge in achieving intimate contact with SERS substrates [16–19]. Consequently, SERS substrates used for efficient detection of VOCs necessitate 3 key attributes: (a) large specific surface area coupled with ample adsorption sites for gas molecules; (b) high SERS activity allowing for sensitive detection of adsorbed trace gas molecules; and (c) good SERS spectral stability enabling the development of practical detection devices [20–25].

To meet the aforementioned requirements, SERS substrates for gas detection based on metal-organic frameworks (MOFs) have attracted widespread interest [26–29]. The selective adsorption of specific molecules, molecular sieve effect, and the capability of effortlessly optimizing electronic structures endow MOFs with promising candidates for SERS applications [30–33]. Fu et al. [34] successfully detected gaseous toluene at concentrations as low as 2.5 parts per million (ppm) using MIL-100(Fe) as SERS substrate and achieved the multiple detection of several VOCs. However, as semiconductor SERS substrates, MOFs still face difficulties in detecting trace amounts of VOCs with concentrations as low as parts per billion (ppb) level, usually requiring the assistance of noble metal nanomaterials. For example, Qiao et al. [35] achieved the trace detection of aldehydes at the ppb level by the superstructure of gold nanoparticles encapsulated by ZIF-8. However, the SERS uniformity of noble metal/MOFs composite structure is poor due to the uneven distribution of “hot spots” caused by the local surface plasmon resonance (LSPR) effect of noble metal nanoparticles [36]. Therefore, it is necessary to develop a construction strategy for designing SPR-free gas detection substrates with high SERS sensitivity and good spectral stability. In comparison with traditional nanomaterials, single-atom materials have lower coordination numbers and unique electronic structures, demonstrating remarkable performance in photochemistry [37–40]. Moreover, the absence of SPR effect in single-atom materials mitigates the issues related to spectral signal uniformity caused by unevenly distributed “hot spot” regions, endowing them with immense potential for SERS sensor applications [41–43]. In our previous research [44], Au single atoms anchored on amorphous C_3_N_4_ nanosheets present remarkable SERS performance based on the single-atom charge transfer (SACT) mechanism, which is promoted by the superior electron delocalization of single atom, and the higher density of state (DOS) near the highest occupied molecular orbital (HOMO) level.

Based on the above analysis, we report a novel single-atom-based SERS sensor (Cu_1_/UiO-66) for VOC detection, which combines the characteristic advantages of MOF and single-atom materials. UiO-66 exhibits a large specific surface area and excellent stability, enabling selective adsorption of benzene-derived VOCs (cancer biomarkers in exhaled breath) through π–π interactions with organic ligands [45,46]. Its intrinsic defects provide anchoring sites for Cu single atoms, considerably enhancing the stability of the metal species. Notably, the adjustable bandgap of UiO-66 confers potential SERS activity. Cu single atoms demonstrate superior chemical stability while offering a cost-effective alternative to noble metals. Moreover, the electronic structure of UiO-66 can be effectively modulated through single-atom loading. Copper single atoms loaded on UiO-66 were successfully synthesized, and SERS limit of detection (LOD) for gaseous toluene is as low as 10 ppb. Remarkably, the signal of VOC adsorption on Cu_1_/UiO-66 can be observed in only 2 min, and it reaches saturation within 20 min. Moreover, it has excellent spectral stability, with a relative standard deviation (RSD) of 6.4%. Enhancement factor (EF) of Cu_1_/UiO-66 can reach 6.71 × 10^9^, representing the most powerful performance among all SPR-free SERS substrates. The Cu single atoms loaded in UiO-66 can profoundly modulate the band structure of UiO-66, enabling efficient coupling with the energy levels of VOC molecules. Furthermore, the high electron delocalization of Cu single atoms drastically promotes the SACT mechanism between Cu_1_/UiO-66 and VOC molecules. The detection limit of Cu_1_/UiO-66 for 2,5-dimethylfuran was as low as 100 ppb in the simulated lung cancer patient exhalations, and its characteristic peak can be clearly distinguished in the mixed gas with toluene. Through the principal component analysis and linear discriminant analysis (PCA-LDA) and hierarchical cluster analysis (HCA) methods of SERS spectra of various gases, it is simulated that the VOCs with similar structures contained in the simulated lung cancer patient exhalations can be identified and distinguished with 100% accuracy. These features substantially amplify the VOC sensing performance of Cu_1_/UiO-66, indicating the great potential of single-atom-based SPR-free SERS substrate for gas detection applications.

## Results and Discussion

### Synthesis of Cu_1_/UiO-66

**Fig. 1.**
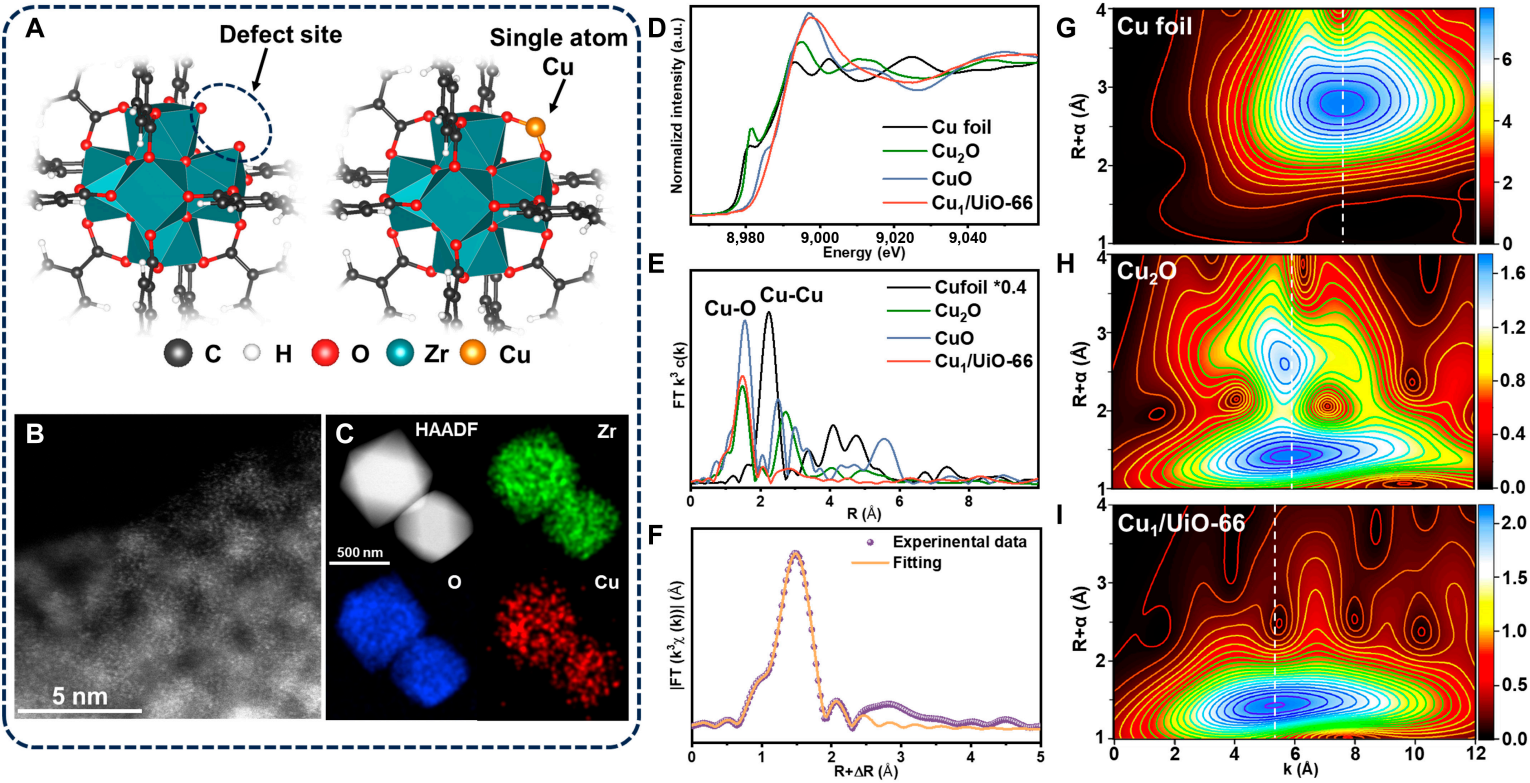
(A) Synthesis schematic of Cu_1_/UiO-66 with the Cu single atom anchored on the defect of the zirconia center in UiO-66. (B and C) HAADF-STEM images of Cu_1_/UiO-66 along with the corresponding EDS mapping of the C, Zr, and Cu K_α_ lines. (D and E) Normalized XANES spectra and FT-EXAFS spectra of Cu_1_/UiO-66 and references at the Cu K-edge. (F) EXAFS fitting curves of Cu_1_/UiO-66 in R-space. (G to I) WT-EXAFS spectra of Cu_1_/UiO-66 and references.

Defective UiO-66 was synthesized via the solvothermal method (Fig. 1A), and its powder x-ray diffraction (PXRD) pattern matched simulations (Fig. S1). The Fourier transform infrared spectroscopy (FTIR) clearly shows the characteristic peaks attributed to UiO-66 (Fig. S2 and Table S1). The N_2_ adsorption isotherm shows a type I isotherm, with a specific surface area of approximately 1,500 m^2^/g, while the pore size distribution diagram shows a regular microporous structure with a pore size of about 0.75 nm (Figs. S3 and S4). The scanning electron microscope (SEM) image revealed UiO-66’s regular octahedral morphology (500 to 800 nm, Fig. S5). The electron paramagnetic resonance spectrum exhibits a distinct peak at *g* = 2.003 (Fig. S6), indicating the presence of unsaturated Zr-O clusters, which can be attributed to ligand defects arising from a missing linker in the UiO-66 framework [47]. Thermogravimetric analysis demonstrated a 48.8% ZrO_2_ residue at 800 °C (Fig. S7), conspicuously exceeding the 44% theoretical value for defect-free UiO-66 [Zr_6_O_4_(OH)_4_(BDC)_6_, BDC = terephthalic acid], confirming the successful synthesis of defective UiO-66. Cu_1_/UiO-66 was synthesized by dispersing UiO-66 in a CuCl_2_·2H_2_O/dimethylformamide (DMF) solution followed by 85 °C overnight heating. Inductively coupled plasma optical emission spectroscopy (ICP-OES) analysis revealed a stable Cu loading of ~0.9 wt%, unaffected by excess CuCl_2_·2H_2_O (Table S2), confirming Cu single-atom saturation. SEM imaging demonstrated preserved octahedral particle morphology before and after Cu incorporation (Fig. S5). PXRD patterns of Cu_1_/UiO-66 (Fig. S1) show excellent agreement with simulated UiO-66 diffractions without any crystalline phases of Cu or CuO*_x_*.

In the high-angle annular dark-field scanning transmission electron microscopy (HAADF-STEM) and energy dispersive spectrometer (EDS) measurements of Cu_1_/UiO-66, the atomic resolution HADDF-STEM images collected from the edge of UiO-66 crystals revealed individual atoms/ions (Fig. 1B). The EDS results confirmed the uniform distribution of Cu elements within the UiO-66 crystal, and no Cu/CuO*_x_* clusters or nanoparticles were observed (Fig. 1C and Fig. S8), which is consistent with the PXRD results. In situ CO adsorption diffuse reflectance infrared Fourier transform spectroscopy (DRIFTS) revealed a distinct peak at 2,158 cm^−1^ in the spectrum of Cu_1_/UiO-66 (Fig. S9), which can be attributed to the stretching vibration of the C=O bond in linearly adsorbed CO, confirming the monoatomic dispersion of Cu [48]. Furthermore, x-ray absorption near-edge structure (XANES) and extended x-ray absorption fine structure (EXAFS) measurements were applied to analyze detailed coordination of Cu atoms in Cu_1_/UiO-66. The normalized XANES spectra clearly showed that the white line intensity of the Cu_1_/UiO-66 sample was stronger than Cu foil and Cu_2_O, approaching the white line intensity of CuO, revealing that the Cu atoms in Cu_1_/UiO-66 exhibited an oxidized state, consistent with the Cu 2p x-ray photoelectron spectroscopy (XPS) spectrum (Fig. 1D and Fig. S10). The Fourier-transform EXAFS (FT-EXAFS) spectra indicated that the Cu atoms in Cu_1_/UiO-66 formed isolated Cu–O coordination structures (Fig. 1E), and no peak corresponding to Cu–Cu bonds were observed at 2.2 Å, suggesting the monatomic distribution of Cu atoms in the material. The EXAFS spectrum of Cu_1_/UiO-66 was further fitted with possible Cu–O coordination structures, and the structural parameters at the Cu K-edge were quantitatively extracted and fitted using the least squares method. The phase-corrected Cu–O interatomic distance of Cu_1_/UiO-66 was approximately 1.92 Å, with a coordination number of approximately 3, consistent with previous reports (Fig. 1F and Table S3) [49]. Additionally, wavelet transform (WT) analysis of the Cu K-edge EXAFS further confirmed the single-atom state of Cu element in Cu_1_/UiO-66. A strong Cu–O coordination was observed in the WT contour plot, with a maximum peak at 5.3 Å. Compared with the WT signals of Cu foil and Cu_2_O, no Cu–Cu coordination was observed. This result further confirmed the single-atom distribution of Cu in Cu_1_/UiO-66 (Fig. 1G to I). In summary, UiO-66 was synthesized and single-atom Cu was successfully loaded, forming Cu_1_/UiO-66.

### SERS performance of Cu_1_/UiO-66

**Fig. 2.**
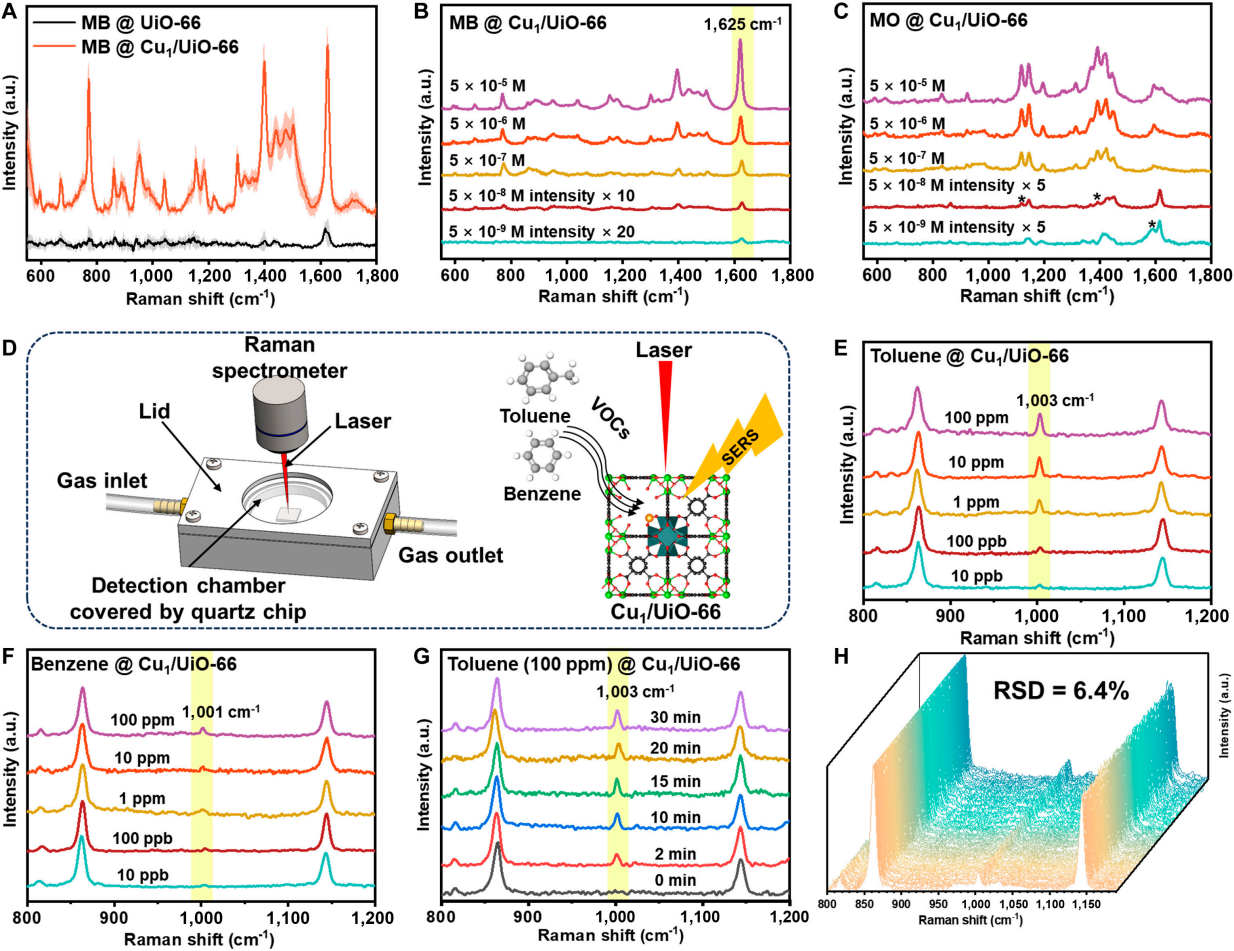
(A) Average Raman spectra of MB with a concentration of 5 × 10^−5^ M adsorbed on UiO-66 and Cu_1_/UiO-66, with the error represented by the shaded area. (B) SERS spectra of MB adsorbed on Cu_1_/UiO-66 with different concentrations, with the characteristic peak at 1,625 cm^−1^ highlighted. (C) SERS spectra of MO adsorbed on Cu_1_/UiO-66 with different concentrations, with characteristic peaks marked by asterisks (*) at low concentrations. (D) Schematic diagram of the SERS device for gas detection. (E) SERS spectra of toluene adsorbed on Cu_1_/UiO-66, with the characteristic peak at 1,003 cm^−1^ highlighted. (F) SERS spectra of benzene adsorbed on Cu_1_/UiO-66, with the characteristic peak at 1,001 cm^−1^ highlighted. (G) Time-dependent SERS spectra of 100 ppm toluene gas flowing over the Cu_1_/UiO-66 substrate, with the characteristic peak at 1,003 cm^−1^ highlighted. (H) Randomly selected 256 SERS curves from 4,000 data points in the SERS spectra of 100 ppm toluene adsorbed on Cu_1_/UiO-66, along with their corresponding RSD values.

The SERS performance of Cu_1_/UiO-66 was evaluated using multiple dye molecules. A 633-nm laser was chosen as it yielded the highest Raman signal for methylene blue (MB) (Fig. S11). Cu_1_/UiO-66 demonstrated obviously enhanced Raman intensity for MB compared to UiO-66 (Fig. 2A). Key vibrational peaks of MB (1,623 cm^−1^) and methyl orange (MO; 1,595 cm^−1^), corresponding to C–C bonds in their aromatic rings [50,51], remained detectable at ultralow concentrations (5 × 10^−9^ M) (Fig. 2B and C; for peak assignments, see Table S4). In contrast, UiO-66 showed no MB detection capability below 10^−6^ M (Fig. S12). Additionally, we observed selective enhancement of nontotally symmetric b_2_ modes at 1,035 cm^−1^ (MB), and 1,392 cm^−1^ (MO), attributed to the Herzberg–Teller contribution [52]. Besides high detection sensitivity, excellent spectral stability is also crucial for SERS detection of VOCs. The stability of SERS signals was evaluated by collecting 4,000 sample points (at 5 × 10^−6^ M) and calculating the RSD based on the characteristic peak intensities at 1,623 cm^−1^ (MB) and 1,595 cm^−1^ (MO). The RSD values were approximately 6.5% for MB and 7.0% for MO, with intensity distributions of these characteristic peaks generally conforming to a normal distribution (Figs. S13 to S16). These results demonstrate that Cu_1_/UiO-66 possesses exceptional SERS performance independently of the SPR mechanism, suggesting its potential for conducting SERS detection of gas samples.

A typical VOC, toluene, was employed to investigate the SERS capability of Cu_1_/UiO-66. Specifically, Cu_1_/UiO-66 was dip-coated onto a silicon slice and then placed in a self-made detection chamber for in situ VOC sensing (Fig. 2D). The Raman spectrum of toluene vapor at a concentration of 1,000 ppm adsorbed on Cu_1_/UiO-66 clearly shows a characteristic Raman peak attributed to toluene at 1,003 cm^−1^, which can be distinguished from the intrinsic Raman characteristic peaks of Cu_1_/UiO-66 (Fig. S17). In the SERS spectra of toluene vapor at a concentration of 100 ppm, the intensity of the characteristic peak of toluene adsorbed on Cu_1_/UiO-66 was higher than that on UiO-66 (Fig. S18). The peak assignment of Cu_1_/UiO-66 and toluene is shown in Table S5. We introduced toluene's water-saturated solution (~ 0.05% wt) into the detection chamber at a concentration of 20,000 ppm, which inherently contained about 10 ppm of toluene. By comparing the SERS spectrum with that of toluene at the same concentration in air devoid of water vapor interference, we found that the signal intensity was not reduced due to the presence of water vapor interference (Fig. S19). To obtain more uniform Raman spectra, the SERS spectra of toluene at different concentrations were normalized using the Raman scattering peak of UiO-66 at 863 cm^−1^ as an internal standard. Through the analysis of the SERS intensity arising from the adsorption of toluene on Cu_1_/UiO-66, we observed that the characteristic peak of toluene at 1,003 cm^−1^ remained clearly discernible even at a toluene concentration as low as 10 ppb (Fig. 2E). The correlation between the toluene concentration and SERS intensity followed an S-shaped function distribution (Fig. S20). Similarly, a superior SERS LOD of 10 ppb was achieved for the detection of gaseous benzene (Fig. 2F). However, UiO-66 failed to observe the characteristic peak of toluene below 15 ppm (Fig. S21).

Additionally, Cu_1_/UiO-66 also exhibited rapid response to VOCs. The characteristic peak of toluene vapor at a concentration of 100 ppm was clearly observed within 2 min of exposure, and adsorption equilibrium was achieved within 20 min (Fig. 2G and Fig. S22). Cu_1_/UiO-66 EF calculation based on the Raman shift of toluene at 1,003 cm^−1^ was revealed as high as 6.71 × 10^9^, which is comparable to that of noble metal SERS materials relying on SPR effect (Table S6). The stability of gas SERS signals was evaluated by randomly collecting and normalizing 4,000 data points each for toluene and benzene adsorbed on Cu_1_/Ui-66 (100 ppm), followed by statistical analysis of their characteristic peak intensities at 1,003 and 1,001 cm^−1^. The results yielded RSD values of approximately 6.4% for toluene and 7.0% for benzene, respectively (Fig. 2H and Figs. S23 to S26). These findings demonstrate that Cu_1_/UiO-66 possesses excellent VOC sensing stability and reliability, making it a highly active SPR-free SERS substrate suitable for quantitative detection. Substrate reuse is crucial for cost-effectiveness in practical applications. Here, we achieved substrate self-cleaning by simply placing the substrate in a thermal vacuum. After Raman spectroscopy detection, the substrate was placed in a vacuum oven at 100 °C for 2 h to facilitate toluene desorption from Cu_1_/UiO-66. As shown in Fig. S26A and B, the characteristic toluene peak at 1,003 cm^−1^ completely disappeared after 2 h. Subsequent re-exposure to toluene gas allowed re-detection of the 1,003 cm^−1^ characteristic peak, demonstrating that Cu_1_/UiO-66 can repeatedly adsorb gaseous toluene samples. As shown in Fig. S27, after 5 adsorption–desorption cycles, distinct toluene Raman peaks remained detectable, and the peak intensity at 1,003 cm^−1^ remained relatively stable across multiple cycles (Fig. S28), indicating excellent cyclic stability of the substrate.

### Enhancement mechanism of Cu_1_/UiO-66

**Fig. 3.**
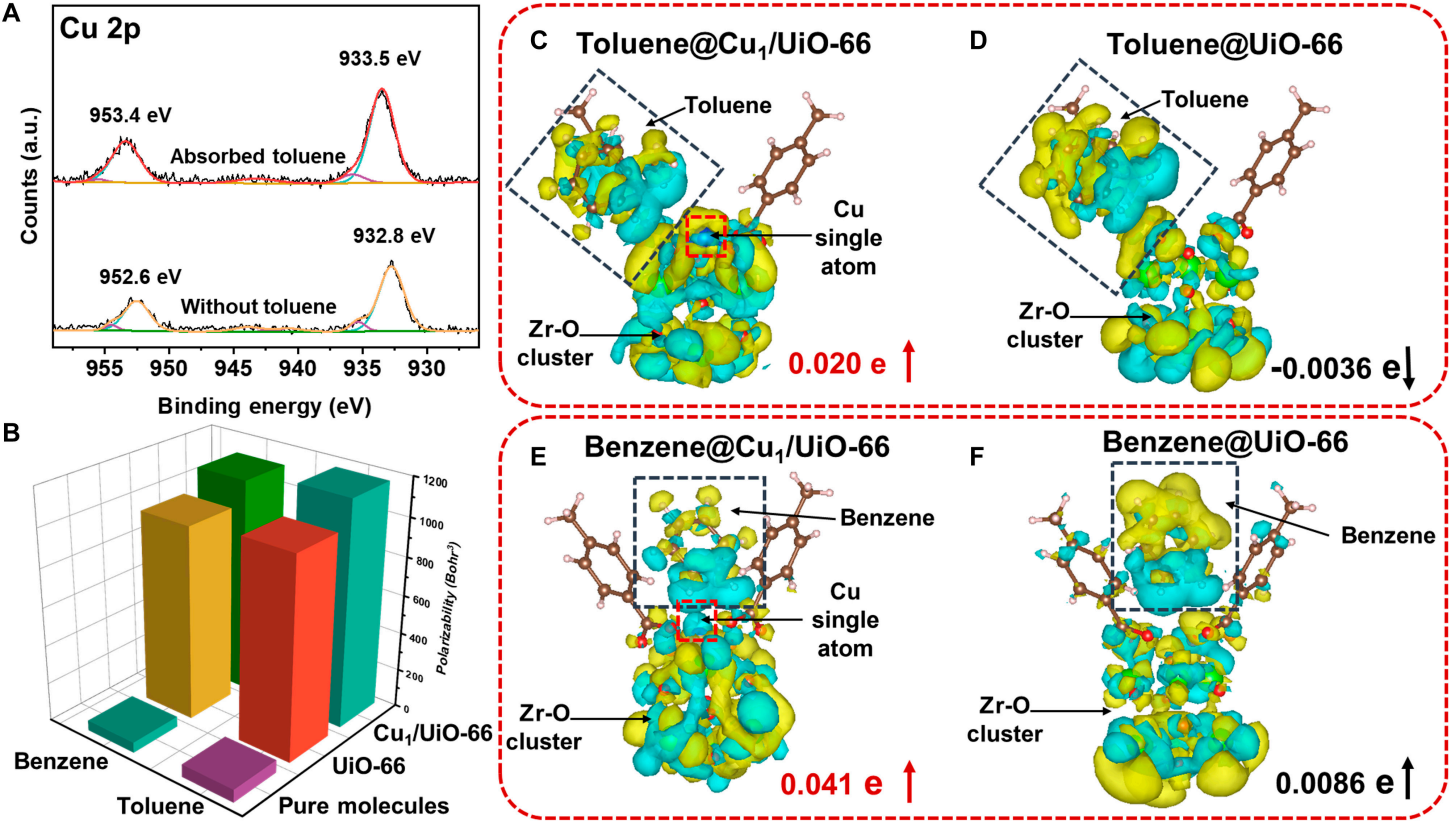
(A) Cu 2p XPS spectra of Cu_1_/UiO-66 before and after being adsorbed with toluene. (B) The calculated polarizability of free state toluene and benzene, UiO-66-benzene/toluene, and Cu_1_/UiO-66-benzene/toluene. The charge difference for the toluene molecule adsorbed onto (C) Cu_1_/UiO-66 and (D) UiO-66, and for the benzene adsorbed onto (E) Cu_1_/UiO-66 and (F) UiO-66; the yellow (blue) distribution illustrates electron accumulation (depletion).

In previous studies by Zhao et al., and our previous report [53–57], the charge transfer (CT) mechanism, stemming from the unique CT process of Cu_1_/UiO-66, has been considered as the primary driver of the observed remarkable SPR-free SERS enhancement. The absence of SPR absorption peak in the ultraviolet–visible diffuse reflection spectra supports the exclusion of electromagnetic mechanism contributions to the enhanced SERS performance of Cu_1_/UiO-66 (Fig. S9). Additionally, absorptions observed in the visible light region underscore its exceptionally strong potential for chemical enhancement. The CT process can be preliminarily confirmed through an analysis of the XPS of Cu single atoms. The Cu 2p XPS spectrum of Cu_1_/UiO-66 exhibits 2 distinct characteristic peaks at 932.8 and 953.4 eV, corresponding to the 2p_3/2_ and 2p_1/2_ of Cu single atoms, respectively. Upon toluene adsorption, the characteristic peaks shifted toward higher binding energies by approximately 0.7 eV, to 933.5 eV for Cu 2p_3/2_ and 953.4 eV for Cu 2p_1/2_, respectively. This shift indicates that after toluene is adsorbed on Cu_1_/UiO-66, there is a strong CT effect from Cu single atoms to toluene molecules (Fig. 3A).

Toluene within UiO-66 was typically adsorbed through π–π interaction [58]. Upon laser excitation, the molecular polarizability was enhanced via an interfacial CT effect, thereby amplifying the molecular Raman scattering cross-section. Density functional theory (DFT) simulations were performed to evaluate the polarizabilities of benzene and toluene in their isolated states and adsorbed states upon UiO-66 and Cu_1_/UiO-66. As shown in Fig. 3B, the polarizabilities of isolated benzene and toluene molecular forms were 54.35 and 66.99 Bohr^3^, respectively. When adsorbed upon UiO-66, π–π interactions govern the absorption process (shown in Figs. S30 and S31), resulting in remarkable increases in their polarizabilities to 1,003.53 and 1,040.08 Bohr^3^, respectively. After the introduction of Cu single atoms, the polarizabilities of the 2 molecules were further increased to 1,169.76 and 1,110.79 Bohr^3^, which contributes to the amplification of the Raman cross-section (Figs. S32 and S33). The incorporation of Cu single atoms effectively elevated the Raman scattering cross-section of the probe molecules adsorbed on them by amplifying their molecular polarizability.

The mechanism of the chemical enhanced process was investigated by calculating the charge difference of valence electron density distributions between Cu_1_/UiO-66 and VOCs, with pure UiO-66 serving as the benchmark. Two probe molecules, benzene and toluene, differing in polarity but similar in structure, were selected for study. As illustrated in Fig. 3C and D, toluene displayed a “pull-electron” behavior with UiO-66, whereas benzene showed a “push-electron” behavior [59]. By incorporation of Cu single atoms, however, both molecules displayed “pull-electron” behaviors, aligning with the XPS analysis. It was observed that Cu_1_/UiO-66 enhanced the transfer of more electrons from the substrate to the molecules compared to UiO-66, with the CT amounts for benzene and toluene increasing from 0.0086 and −0.0036 e to 0.041 and 0.020 e, respectively. The enhanced CT capability substantially boosts the activity of the Cu_1_/UiO-66’s SERS effect (Fig. 3E and F).

**Fig. 4.**
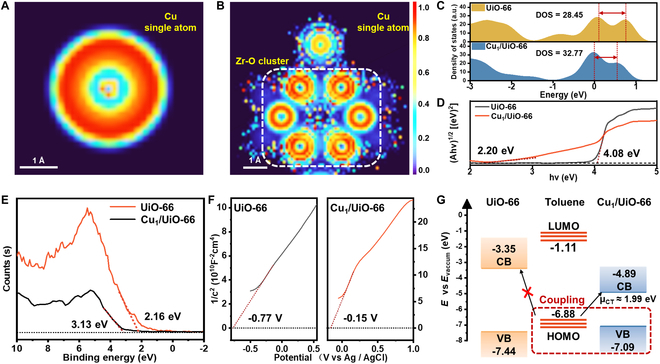
Two-dimensional ELF diagram of (A) free state Cu single atom and (B) Cu single atom adsorbed on the Zr-O cluster of UiO-66. (C) Calculated total density of states (TDOS) of Cu_1_/UiO-66 and UiO-66. (D) Tauc plot of UiO-66 and Cu_1_/UiO-66. (E) X-ray photoelectron spectroscopy (XPS) valence band spectra of UiO-66 and Cu_1_/UiO-66. (F) The Mott–Schottky plot of UiO-66 and Cu_1_/UiO-66. (G) Charge transfer pathways of toluene with UiO-66 and Cu_1_/UiO-66 under excitation light at 633 nm.

The enhancement in CT quantity is likely intimately associated with the electronic delocalization of Cu single atoms anchored on UiO-66. To reveal the role of electron delocalization from Cu single atoms in the CT process, post-DFT electron localization function (ELF) analyses were performed. As shown in Fig. 4A, the ELF contours of Cu single atoms exhibit higher values, indicating the presence of more localized electrons. However, upon Cu atom attachment to UiO-66, the Cu valence electrons were attracted by Zr-O clusters, leading to reduced ELF contour values in the vicinity of the Cu single atom (Fig. 4B). The cloud of valance electrons exhibited a fragmented trend, which can be reflected by the fragmented spots around the outer ring of Cu single atoms in the ELF images in Fig. 4B. The ELF analyses suggest that Cu single atoms loaded on UiO-66 possessed superior electron delocalization and were more prone to participating in CT processes. This enhanced electron delocalization facilitated easier electron gain or loss during interactions with organic probe molecules. Therefore, the delocalized electrons directly participate in the SERS enhancement process through the SACT mechanism, thereby increasing the overall amount of the CT process.

DFT calculations revealed narrowed bandgaps and enhanced DOS near the Fermi level in Cu_1_/UiO-66 compared to pristine UiO-66. The maximum DOS value for UiO-66 was approximately 28.5, while for Cu_1_/UiO-66, it was approximately 32.77 (Fig. 4C). This suggests that the incorporation of Cu single atoms grants Cu_1_/UiO-66 a remarkable advantage over UiO-66 in facilitating CT from the substrate to probe molecules. The Tauc plot in Fig. 4D demonstrates a narrowed bandgap of 2.20 eV in Cu_1_/UiO-66 compared to the 4.08 eV of pristine UiO-66 following Cu single-atom loading. Additionally, XPS valence band spectra and Mott–Schottky plots (Fig. 4E and F) revealed band structure differences between Cu_1_/UiO-66 and UiO-66. The XPS VB edge positions from Fermi level (*E*_F_) were 3.13 eV (UiO-66) versus 2.16 eV (Cu_1_/UiO-66), while Mott–Schottky-derived *E*_F_ measured −4.31 and −4.93 eV, respectively. Combined analysis established their energy levels: UiO-66 (CB = −3.35 eV, VB = −7.44 eV) vs. Cu_1_/UiO-66 (CB = −4.89 eV, VB = −7.09 eV), as summarized in Fig. 4G. The HOMO and lowest unoccupied molecular orbital (LUMO) of toluene molecule were calculated to be −6.88 and −1.11 eV, respectively [34]. Obviously, toluene adsorption on Cu_1_/UiO-66 triggers HOMO-VB coupling, mediating CT process from the HOMO level to the CB of Cu_1_/UiO-66 under 633 nm laser excitation (~1.96 eV photon energy). Therefore, introducing Cu single atoms enhances substrate-molecule CT while modifying the band structure of UiO-66. The markedly increased CT probability amplifies molecular polarizability, thereby strengthening Raman signal intensity.

### Detection and identification of simulated exhalations

**Fig. 5.**
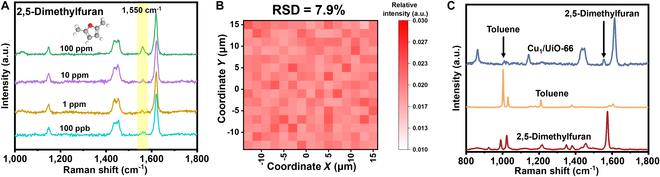
(A) SERS spectra of 2,5-dimethylfuran with different concentrations adsorbed on Cu_1_/UiO-66, with the characteristic peak at 1,550 cm^−1^ highlighted. (B) Raman mapping image represents signal intensities of 2,5-dimethylfuran at the Raman shift of 1,550 cm^−1^ within an area of 30 × 30 μm. (C) SERS spectra of 100 ppm mixture of toluene and 2,5-dimethylfuran adsorbed on Cu_1_/UiO-66.

Trace amounts of 2,5-dimethylfuran, often found in the exhaled breath of cancer patients and recognized as a biomarker, can effectively facilitate early screening and diagnosis of lung cancer through its detection [60–62]. Therefore, Cu_1_/UiO-66 enabled trace detection of lung cancer-associated 2,5-dimethylfuran in simulated exhalations. As shown in Fig. 5A, the characteristic peak at 1,550 cm^−1^ could be observed at a low concentration of 100 ppb when it was adsorbed onto Cu_1_/UiO-66. Furthermore, a randomly selected area of 30 μm × 30 μm with 256 points was subjected to Raman mapping tests. By analyzing the intensity of the characteristic peak located at 1,550 cm^−1^, it was found that the RSD was 7.9%, demonstrating excellent SERS spectral stability (Fig. 5B). Additionally, it is possible that other VOC molecules may be presented in the simulated exhaled breath. The VOCs present in the exhaled breath of cancer patients may interfere with one another, leading to challenges in accurate SERS detections. To explore the recognition capability of the constructed SERS sensor for a range of VOCs, a solution of toluene (100 ppm) and 2,5-dimethylfuran (100 ppm) in a 1:1 ratio was prepared. As shown in Fig. 5C, the characteristic peak of toluene at 1,003 cm^−1^ could be clearly distinguished from the characteristic peak of 2,5-dimethylfuran at 1,550 cm^−1^, which indicated the excellent anti-interference detection capacity of Cu_1_/UiO-66.

**Fig. 6.**
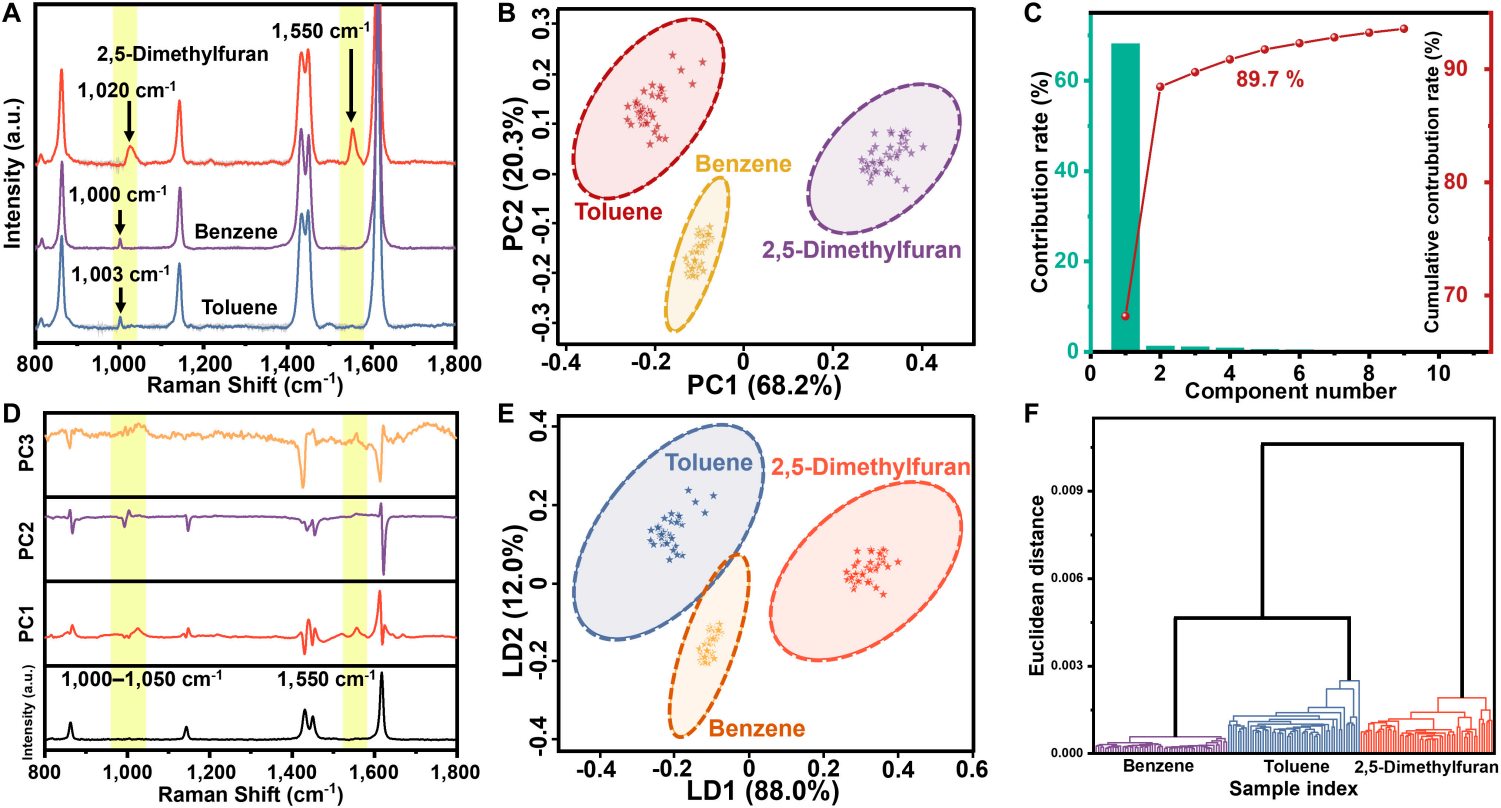
(A) Average SERS spectra of 100 ppm VOCs absorbed on Cu_1_/UiO-66 highlighting characteristic peaks: 1,020/1,550 cm^−1^ (2,5-dimethylfuran), 1,001 cm^−1^ (benzene), and 1,003 cm^−1^ (toluene), with shaded error bars. (B) 2D PCA score plot of VOCs@Cu_1_/UiO-66 SERS data showing class segregation with 95% confidence ellipses color-coded by VOC type. (C) PCA scree plot (SERS data) displaying variance explained by PC1 to PC10 and cumulative variance (secondary axis). (D) PCA loading plot showing spectral variable contributions to PC1 to PC3, with wavenumber annotations highlighting discriminant regions. (E) LDA score plot projecting samples onto LD1/LD2, with 95% confidence ellipses. (F) HCA dendrogram (Ward's linkage and Euclidean distance) of SERS profiles showing cluster formation at dissimilarity threshold X, color-coded by VOC class.

Given the high degree of similarity observed in the SERS spectra of 3 VOC gas molecules when adsorbed on Cu_1_/UiO-66 at low concentrations (Fig. 6A), we employed the PCA-LDA for the purpose of further classification and identification of their “fingerprint” SERS spectra. To assess the performance of the analytical model, we performed 10 independent experiments, in each of which 50 SERS spectra were randomly selected from the 54 available for each sample type for PCA-LDA. The 10-fold cross-validation accuracy was 100% in each experiment (Fig. S34). As shown in the PCA score plot in Fig. 6B, the 3 VOCs can be clearly distinguished. The scree plot in Fig. 6C displays the relationship between the principal components of the SERS spectra and their eigenvalues, thereby demonstrating the proportion of total variance accounted for by each principal component. The first 3 principal components account for 89.7% of the total variance, effectively explaining the original spectral characteristics. Figure 6D shows the loading plot of PC1, PC2, and PC3, indicating that the Raman peaks at 1,000 to 1,020 cm^−1^ and 1,550 cm^−1^ can serve as key spectral features for differentiating the 3 VOCs.

As shown in Fig. 6E, the utilization of the first 3 principal components obtained from PCA for LDA processing resulted in the clear segregation of the 3 gas molecules into distinct clusters. Additionally, the confusion matrix obtained confirmed that the classification accuracy of the multi-dimensional LDA analysis for the 3 gas molecules was 100% (Fig. S35). Moreover, these SERS spectra may be subjected to clustering analysis through the HCA. As illustrated in Fig. 6F, the horizontal lines in the dendrogram indicate which samples and categories are linked, while the vertical lines represent the distances between linked categories. The HCA results have successfully identified 3 distinct clusters corresponding to benzene, toluene, and 2,5-dimethylfuran. This method allows for a precise distinction between the subtle differences present in their SERS spectra. Each compound's unique spectral features are clustered separately, enabling clear differentiation and analysis. Therefore, Cu_1_/UiO-66 SERS platform holds great potential for sensor applications in the detection and identification of VOCs.

## Conclusion

In summary, the SPR-free Cu_1_/UiO-66 substrate enables stable and reproducible trace VOC detection via an in situ device. It achieves a toluene LOD of 10 ppb, an EF of 6.71 × 10^9^, and an RSD of 6.4%, outperforming current SPR-free substrates and rivaling noble metal SERS materials. Moreover, gaseous VOCs enrichment and identification can be done in 20 min, showing great spectral stability and quick response. The polarizability of adsorbed molecules can be considerably amplified after being absorbed on Cu_1_/UiO-66 due to the high electronic delocalization of Cu single atom and higher DOS at the Fermi level of Cu_1_/UiO-66, which will promote the CT process effectively. In the detection of exhaled breath simulating that of lung cancer patients, 2,5-dimethylfuran concentrations as low as 100 ppb can be successfully detected, and multiple VOCs can be clearly distinguished and identified through the PCA-LDA and HCA methods, indicating that Cu_1_/UiO-66 has promising application prospects in real-time gas detection and early cancer screening.

## Materials and Methods

### Study design

The objective of this study is to investigate the gas SERS sensing performance, enhancement mechanism, and potential applications of Cu_1_/UiO-66. Sample gases containing VOCs were generated using a VOC generator (details provided in the Supplementary Materials).

### Reagents

Terephthalic acid and benzene were purchased from Innochem Co., Ltd. Zirconium chloride, copper chloride dihydrate, glacial acetic acid, methanol, N,N-DMF, MB, MO, and toluene were purchased from Aladdin Co., Ltd. All chemicals were of analytical grade and required no further purification before use.

### Synthesis of UiO-66

The synthesis of UiO-66 and Cu_1_/UiO-66 was based on the report by Abdel-Mageed et al. [63] with slight modifications. Typically, zirconium chloride (167 mg) and terephthalic acid (125 mg) were dissolved in 50 ml of DMF through ultrasonic agitation for 30 min. Subsequently, 3.5 ml of glacial acetic acid was added and thoroughly mixed. The solution was then transferred to a 100-ml Teflon-lined hydrothermal autoclave and reacted at 120 °C for 24 h. After the autoclave had cooled to room temperature, the sample was washed 5 times with DMF and methanol, respectively. The sample was then soaked in 50 ml of methanol overnight, followed by centrifugation and being dried under dynamic vacuum at 120 °C.

### Synthesis of Cu_1_/UiO-66

In a 10-ml glass vial, 270 mg of copper chloride dihydrate (CuCl_2_ 2H_2_O) was dissolved in 4.5 ml of DMF, followed by the addition of 300 mg of UiO-66. After 5 min of ultrasonic agitation for uniform dispersion, the vial was sealed and placed in an oven at 85 °C for 24 h. The product was washed 5 times with DMF and methanol, respectively, and then soaked in 30 ml of methanol overnight. After centrifugation, the product was finally dried under dynamic vacuum at 120 °C for 12 h.

### PCA-LDA calculation

The PCA-LDA implementation code was written using Python, and subsequently optimized and reviewed through artificial intelligence (AI) techniques to enhance computational accuracy. The AI contributes to software development by assisting in code generation, offering suggestions for patterns, syntax, and logic based on the context, which is beneficial for rapid prototyping and learning best practices. Additionally, it enhances code quality by detecting potential bugs and anomalies and suggesting fixes, saving time and effort in debugging and maintenance processes.

In this study, we employed LDA to address a 3-class problem with an impressive outcome. Through rigorous *K*-fold cross-validation (*K* = 5 and *K* = 10), each repeated 10 times, our model achieved a remarkable 100 % accuracy rate. This result underscores the efficacy of LDA in distinguishing between classes and its robust performance across different validation settings, highlighting its potential in similar classification tasks.

### DFT calculations

The MOF substrate was taken from the UiO-66 and Cu_1_/UiO-66 unicell. The Cu atom was put on the defected dimer oxygen atoms. The benzene and toluene molecules were put around the Cu atom, respectively. The geometry optimization calculations for the adsorption process were performed based on the DFT with Perdew–Burke–Ernzerhof (PBE) functional with Gaussian 16 code with the substrate position fixed [64–66]. The lanl2dz basis functions are applied to the system [67,68]. Then, the Raman data were collected by the vibration behavior for the benzene and toluene molecules on the UiO-66 and Cu_1_/UiO-66 substrate. Finally, the charge transform behavior between the benzene/toluene molecules and the UiO-66/Cu_1_/UiO-66 were calculated, respectively.

### DOS DFT calculation

DFT calculations were performed to understand the DOS change for the UiO-66 and Cu_1_/UiO-66 substrate [69–71]. The PBE functional within the generalized gradient approximation (GGA) method and Broyden–Fletcher–Goldfarb–Shanno update scheme [72] was utilized to explore the optimizing structure with a minimum energy. The energy cutoff of electron wave function is set as 300 eV [73]. The UiO-66 and Cu_1_/UiO-66 substrates were placed into simulation boxes set to 20 × 20 × 20 Å^3^, respectively.

### RSD calculation

For instance, toluene, according to the RSD calculation formula (Eq. 1):

$$c_{v}=\frac{\sigma }{\mu }$$ (1)

The coefficient of variation $c_{v}$ is calculated as the ratio of the standard deviation (σ) to the average intensity (μ). Given that $c_{v}=\frac{\sigma }{\mu }=\frac{0.0183}{0.2836}\approx 6.45\%<10\%$, it suggests a high degree of uniformity, stability, and reproducibility in the SERS signal [74].

### EF calculation

The EF was calculated through Eqs. 2 to 5 [34]:

$$\begin{array}{c} \mathrm{EF}=\frac{\frac{I_{\text{SERS}}}{N_{\text{SERS}}}}{\frac{I_{\text{bulk}}}{N_{\text{bulk}}}}=\frac{I_{\text{SERS}}N_{\text{bulk}}}{I_{\text{bulk}}N_{\text{SERS}}} \end{array}$$ (2)

$$\begin{array}{c} N_{\text{SERS}}=\frac{\frac{\rho V_{\text{toluene SERS}}}{M}N_{A}hA_{\text{Raman spot}}}{V_{\text{container}}} \end{array}$$ (3)

$$\begin{array}{c} \begin{array}{c} N_{\text{bulk}}=\frac{\frac{\rho V_{\text{toluene}}}{M}N_{A}hA_{\text{Raman spot}}}{V_{\text{toluene}}}=\frac{\rho hA_{\text{Raman spot}}N_{A}}{M} \end{array} \end{array}$$ (4)

$$\begin{array}{c} \begin{array}{c} \mathrm{EF}=\frac{I_{\text{SERS}}V_{\text{container}}}{I_{\text{bulk}}V_{\text{toluene SERS}}}=\frac{I_{\text{SERS}}}{I_{\text{bulk}}}\times 4.22\times 10^{10}=6.71\times 10^{9} \end{array} \end{array}$$ (5)

*I*_SERS_ and *I*_bulk_ represent the intensities of the selected Raman peak (1,003 cm^−1^) in the SERS and normal Raman spectra, respectively. *N*_SERS_ and *N*_bulk_ refer to the average number of molecules in the focal spot area for the evaporated and liquid toluene. *V*_container_ is the volume of gas flowing through the detection chamber (1 l/min for 30 min). Since the volume of the detection chamber is considerably smaller than the total volume of gas, it can be disregarded. *V*_toluene SERS_ represents the liquid toluene content in 30 l of air at 10 ppb (0.000712 μl); *A*_Raman_ is the area of the laser spot (1.5 μm in diameter), *N*_A_ is the Avogadro constant, *h* is the depth of the focus of the laser beam (μm), and *M* and *ρ* are the molecular weight and density of toluene (g/cm^3^), respectively.

## Data Availability

All data are available in the main text or the Supplementary Materials.
