# Supplementary material for: Single-Atom Cu Anchored on a UiO-66 Surface-Enhanced Raman Scattering Sensor for Trace and Rapid Detection of Volatile Organic Compounds
Source: Research (Wash D C). 2025 Aug 21;8:0841. doi: 10.34133/research.0841 (PMC12368966; doi:10.34133/research.0841)
Supplement: Supplementary 1 — Supporting text Figs. S1 to S35 Tables S1 to S6 [file research.0841.f1.docx]

Supporting Information for

**Single-Atom Cu Anchored on UiO-66 Surface-Enhanced Raman Scattering Sensor for Trace and Rapid Detection of Volatile Organic Compounds**

Yuening Wang ^†^, Xiangyu Meng ^†^, Wenxiong Shi ^†^, Yujiao Xie ^†^, Aochi Liu ^†^, Lei Xu, Lin Qiu^*^, Xiaoyu Song, Mingjian Zhang, Jiahao Zhang, Jian Yu, Aiguo Wu^*^, Xiaotian Wang^*^ and Jie Lin^*^

Email:

qiulin@ustb.edu.cn

aiguo@nimte.ac.cn

wangxt@buaa.edu.cn

linjie@nimte.ac.cn

Table of Contents

[Supporting text 1](#_Toc203150377)

[Structural characterization of UiO-66 and Cu_1_/UiO-66 3](#_Toc203150378)

[SERS performance of UiO-66 and Cu_1_/UiO-66 16](#_Toc203150379)

[Characterization and Simulation of the SERS Enhancement Mechanism 37](#_Toc203150380)

[Statistical analysis of mixed gas SERS detection 42](#_Toc203150381)

Supporting text

**Characterizations**

The morphologies of the sample were observed by field emission scanning electron microscopy (FE-SEM) measurements, a Quanta 250 FEG was used with an accelerating voltage of 20 kV. For PXRD spectra were collected by SHIMADZU Xlab6000 X-ray diffractometer with Cu Kα X-ray source (λ = 1.5418 Å) with a scan rate of 2 °/min. The FT-IR spectra were collected by Bruker TENSORN II FT-IR spectrometer. The TGA curves were recorded by a synchronous thermal analyzer (NETZSCH TG209F 1Libra) under nitrogen atmosphere, and the temperature range was 40-800 ℃, and the heating rate was 5 ℃/min. The nitrogen adsorption isotherm, pore size distribution, and specific surface area were analyzed using an ipore 600 analyzer, and degassed at 120 ℃ for 12 h before N_2_ adsorption–desorption.

TEM and high angle annular dark field scanning transmission electron microscopy (HADDF-STEM) were carried on HITACHI Talos transmission electron microscope with the acceleration voltage of 200 kV. X-ray photon spectroscopy (XPS) tests were taken in an Thermo Scientific K-Alpha X-ray Photoelectron Spectrometer using an Al Kα X-ray source (1486.6 eV). UV-Vis DRS spectra were collected by SHIMADZU UV-3600 plus. The Raman spectra were acquired with a Jobin Yvon Raman spectrometer model HR800 with the excitation wavelength of 633 nm He-Ne laser under room temperature. The area of laser spot was 1.5 μm^2^ with a 50x objective lens. The laser power was about 5.1 mW, and all tests were maintained the same integration time (10 s). In the VOC SERS detection, firstly, 12 μL of Cu_1_/UiO-66 methanol solution (20 mg/mL) was dropped onto a 7 $\times$7 mm monocrystalline silicon wafer and then dried in a vacuum at 120 ℃. The dried wafer was placed in a self-made gas detection box with a quartz window. VOC vapor was generated by a VOC gas generator (Suzhou AROS Environmental Generators Co., Ltd.) and introduced into the box, achieving in-situ VOC sensing through the quartz window on top.

XAFS measurements: The results were collected in the transmission mode at the XAFCA beamline station in the Singapore Synchrotron Light Source (SSLS). The storage ring of the SSLS was operated at 7.0 GeV with the current below 200 mA.

To evaluate the dye molecule SERS performance, Cu₁/UiO-66 was dispersed in anhydrous methanol (0.1 mg/mL). Upon mixing with dye molecules, 100 μL of Cu₁/UiO-66 dispersion was combined with 100 μL of dye solution at specific concentrations (1×10⁻4 to 1×10⁻8 M), followed by 30 minutes of ultrasonic treatment for uniform mixing and 12 hours of standing, yielding final dye concentrations of 5×10⁻⁵ ~ 5×10⁻⁹ M. For SERS testing, 10 μL of the mixture was drop-cast onto a 7 mm×7 mm single-crystal silicon wafer, which was dried in a 60°C vacuum oven prior to measurement. The integration time was set to 10 seconds with a 633 nm laser wavelength.

The Mott-Schottky plot was measured as follows: 1mg Cu_1_/UiO-66 was dispersed into a mixture of 350 μL deionized water and 150 μL ethanol, and then 15 μL Nafion perfluorinated resin solution (5 % wt) was added as a binder and sonicated for 5 minutes to ensure uniform mixing. The mixed solution was uniformly coated on the surface of Indium-Tin-Oxide (ITO) conductive glass to form a 1 cm × 1 cm square coating film as the working electrode. Mott-Schottky measurements for the samples were conducted in a 0.5 M Na_2_SO_4_ aqueous electrolyte, employing an Ag/AgCl reference electrode and a graphite counter electrode. Impedance-potential curve measurements were conducted using a CHI660E electrochemical workstation (Shanghai Chenhua) with the following parameters: voltage range of -0.6 V to 1 V, frequency of 1000 Hz, and quiet time of 2 seconds.

Structural characterization of UiO-66 and Cu_1_/UiO-66


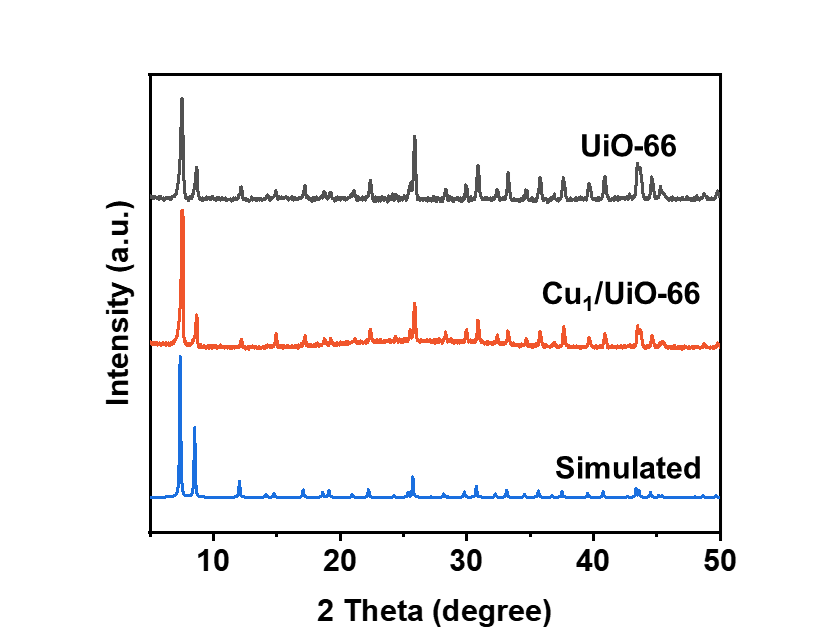


**Figure S1.** PXRD spectra of UiO-66, Cu_1_/UiO-66 and simulated PXRD spectrum of UiO-66.


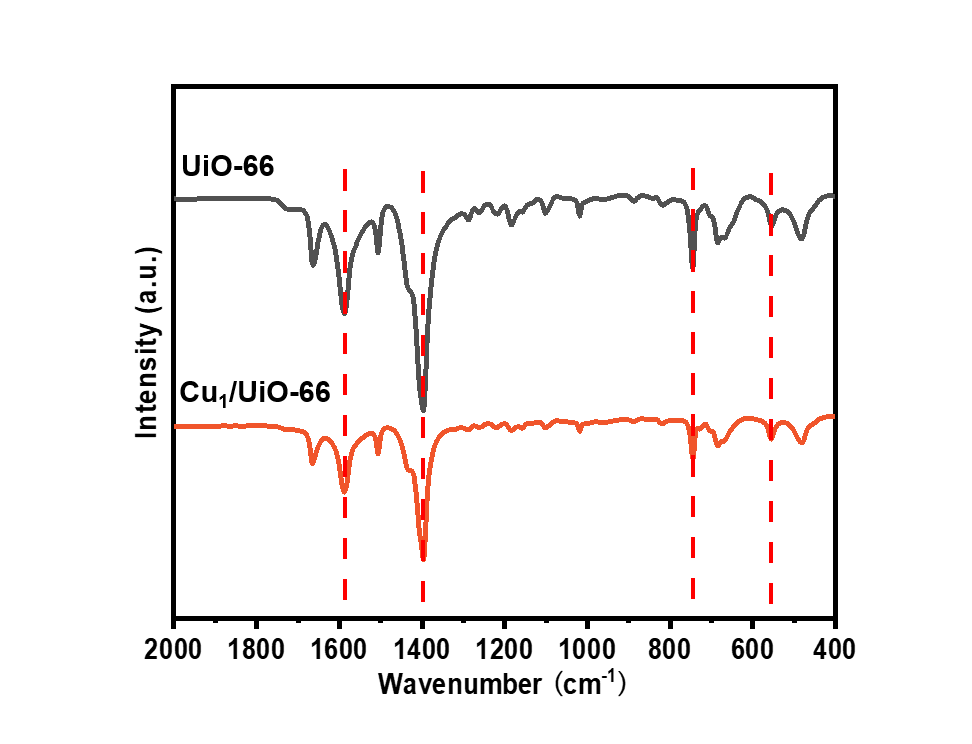


**Figure S2.** FT-IR spectra of UiO-66 and Cu_1_/UiO-66.

**Table S1.** Peak assignment of the FT-IR spectra

| Wavenumber / cm^-1^ | Assignment |
| --- | --- |
| 1589 | O=C-O asymmetric stretching vibration |
| 1506 | Benzene ring C=C vibration |
| 1396 | O=C-O symmetric stretching vibration |
| 1185 / 1101 / 1017 | Zr-O stretching vibration |
| 818 / 748 / 665 | Terephthalic acid OH and C-H vibrations |
| 667 | μ_3_-O telescopic vibration |
| 556 | Zr(O-C) telescopic vibration |
| 480 | μ_3_-OH telescopic vibration |

**Figure S3.** Nitrogen adsorption isotherm of UiO-66 and Cu_1_/UiO-66.

**Figure S4.** Micropore size distribution diagram of UiO-66 and Cu_1_/UiO-66.


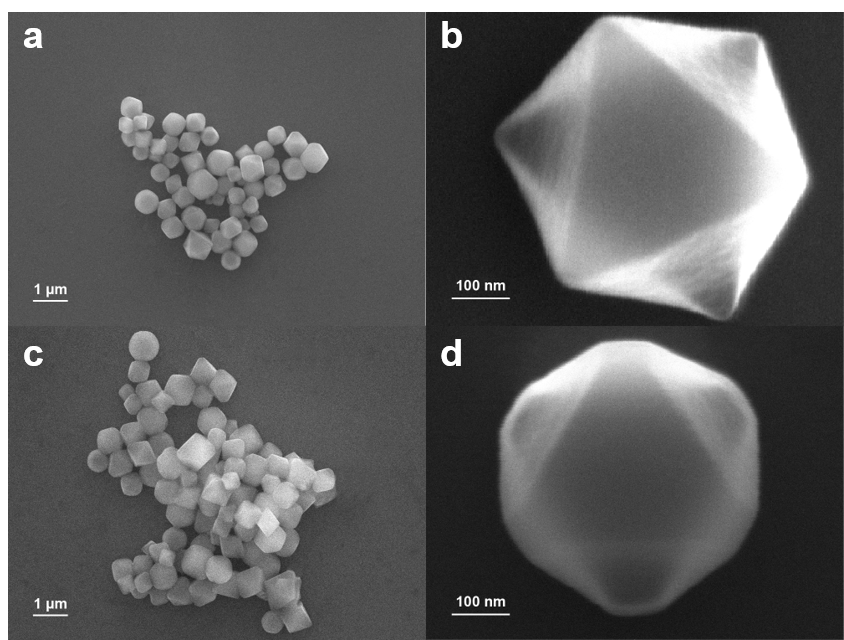


**Figure S5.** SEM images of UiO-66 (a - b) and Cu_1_/UiO-66 (c - d).

**Figure** **S6.** EPR spectrum of UiO-66.


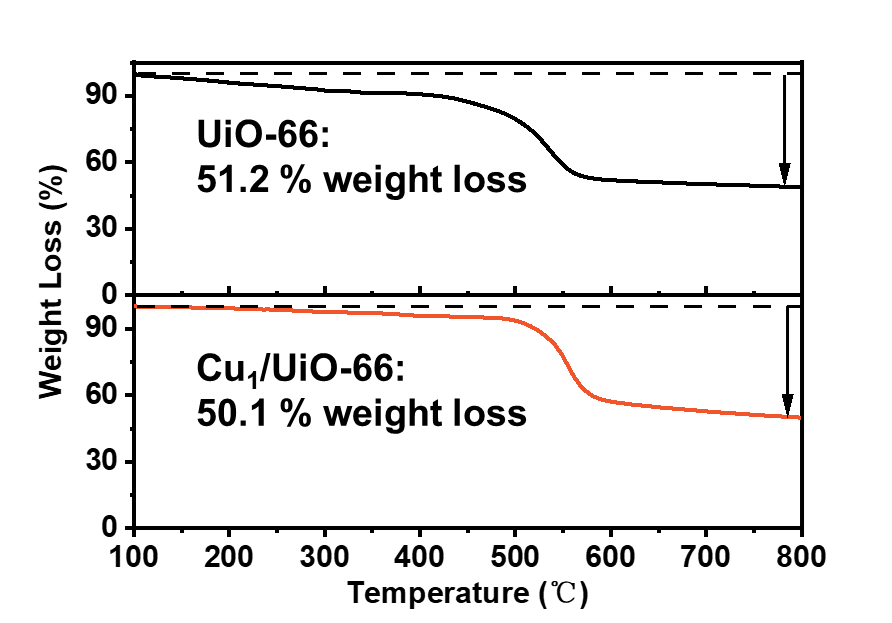


**Figure** **S7.** TGA trace of UiO-66 (up) and Cu_1_/UiO-66 (down) under N_2_ flow.

**Table S2.** Cu content in Cu/UiO-66 synthesized with different amounts of CuCl_2_ 2H_2_O.

| Sample | Cu content / wt % |
| --- | --- |
| 300 mg UiO-66 + 270 mg CuCl_2_ 2H_2_O | 0.95 |
| 300 mg UiO-66 + 473 mg CuCl_2_ 2H_2_O | 1.14 |

**Table S3.** Structural parameters extracted from the Cu K-edge EXAFS fitting. (S02=0.85 from Cu foil).

| Sample | Path | CN | R(Å) | σ^2^(10^-3^Å^2^) | | ΔE_0_(eV) | R factor |
| --- | --- | --- | --- | --- | --- | --- | --- |
| Cu_1_/UiO-66 | Cu-O | 3.4 | 1.92 | | 7.1 | 3.7 | 0.004 |
| Cu foil | Cu-Cu | 12* | 2.53 | | 8.1 | 3.7 | 0.002 |

S_0_^2^ is the amplitude reduction factor; CN is the coordination number; R is interatomic distance (the bond length between Ru central atoms and surrounding coordination atoms); σ^2^ is Debye-Waller factor (a measure of thermal and static disorder in absorber-scatterer distances); ΔE_0_ is edge-energy shift (the difference between the zero kinetic energy value of the sample and that of the theoretical model). R factor is used to value the goodness of the fitting.

* This value was fixed during EXAFS fitting, based on the known structure.

Error bounds that characterize the structural parameters obtained by EXAFS spectroscopy were estimated as N ± 20%; R ± 1%; σ^2^ ± 20%; ΔE_0_ ± 20%.


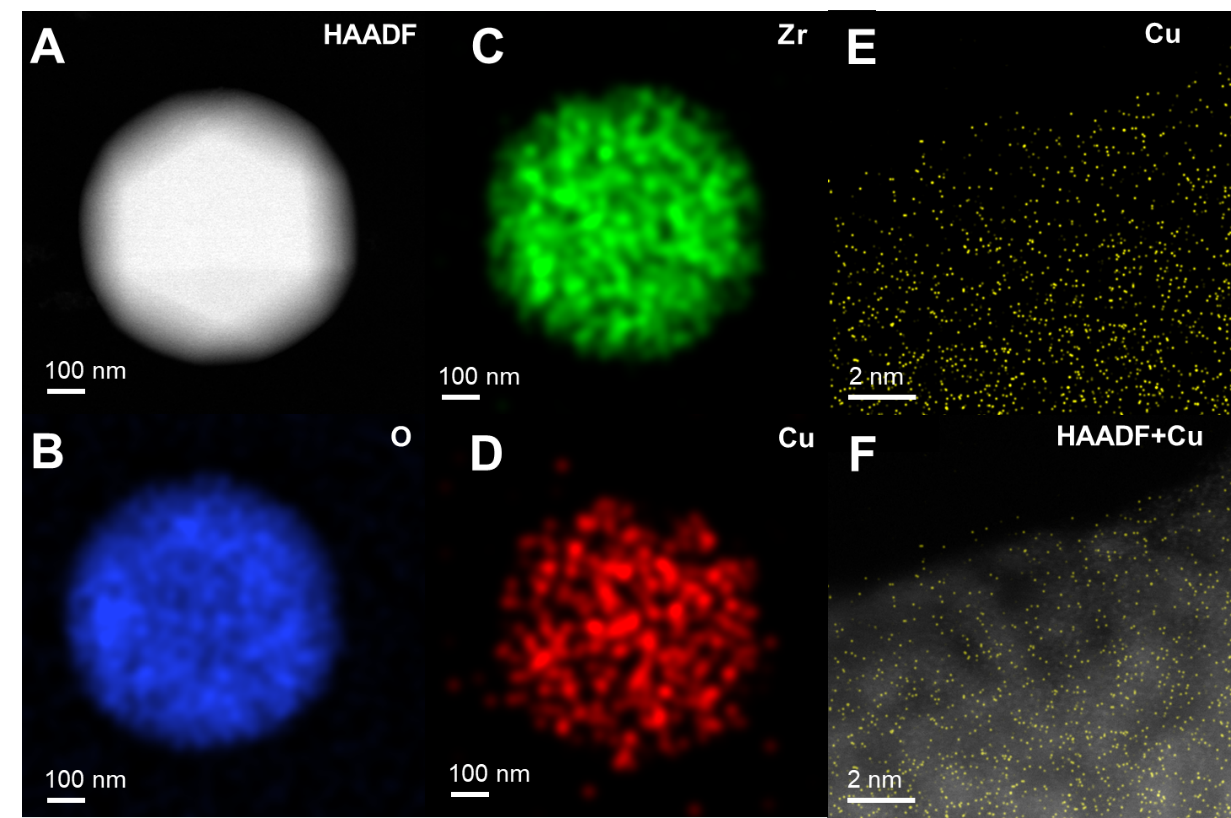


**Figure S8.** (A)HAADF-STEM image of Cu_1_/UiO-66; (B-D) EDS mapping images of Zr, O, and Cu K_α_ lines, respectively; (E) High resolution EDS mapping image of Cu K_α_ lines; (F) High-resolution HAADF and Cu Kα EDS overlay image.


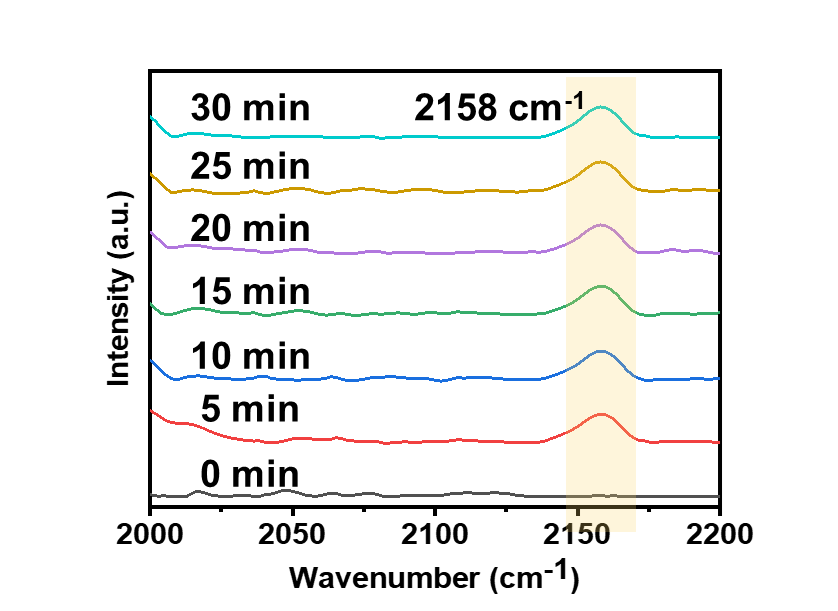


Figure S9. DRIFTS spectra of Cu₁/UiO-66 under different CO adsorption times.


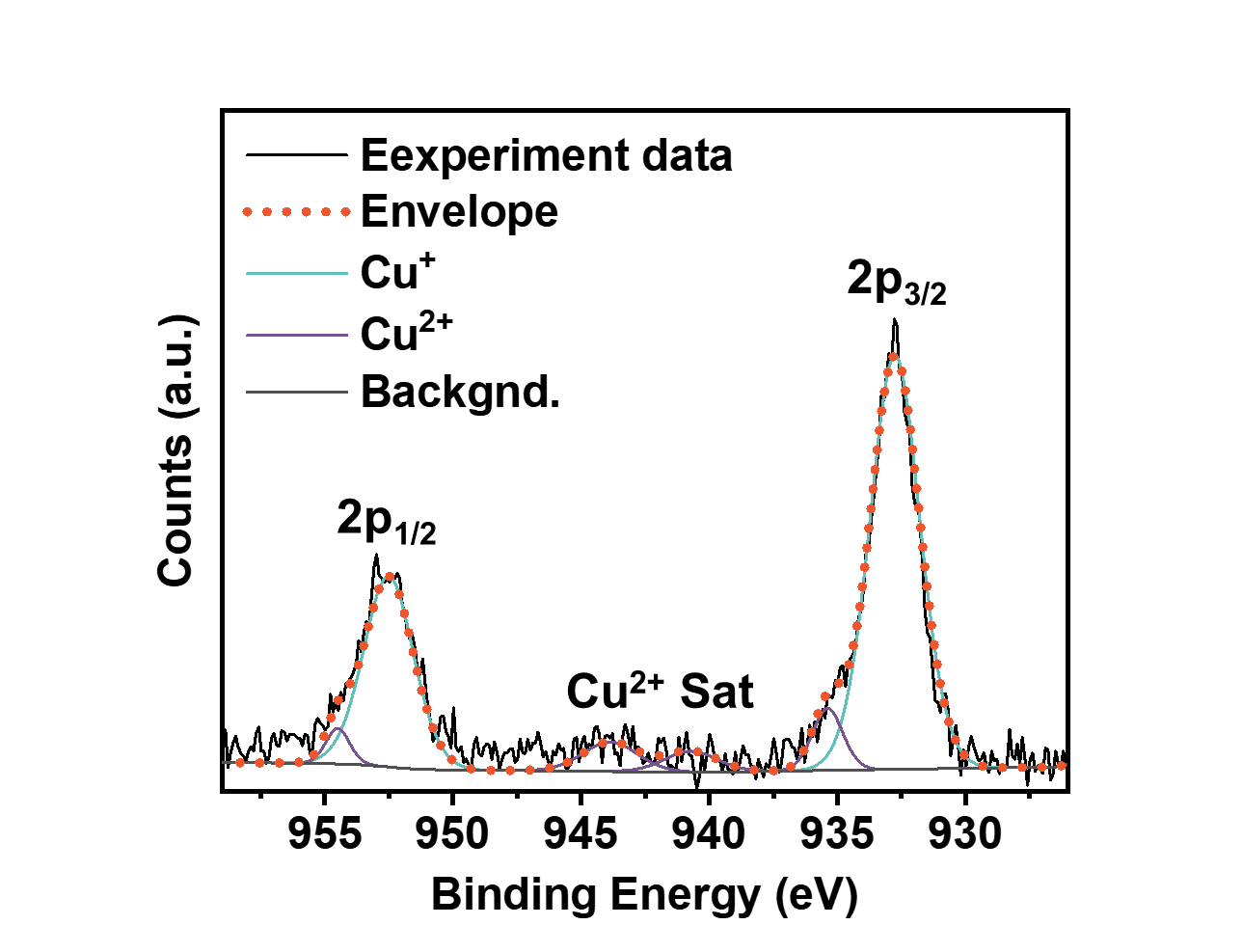


**Figure S10.** XPS spectrum of Cu_1_/UiO-66 in the Cu 2p region.

SERS performance of UiO-66 and Cu_1_/UiO-66


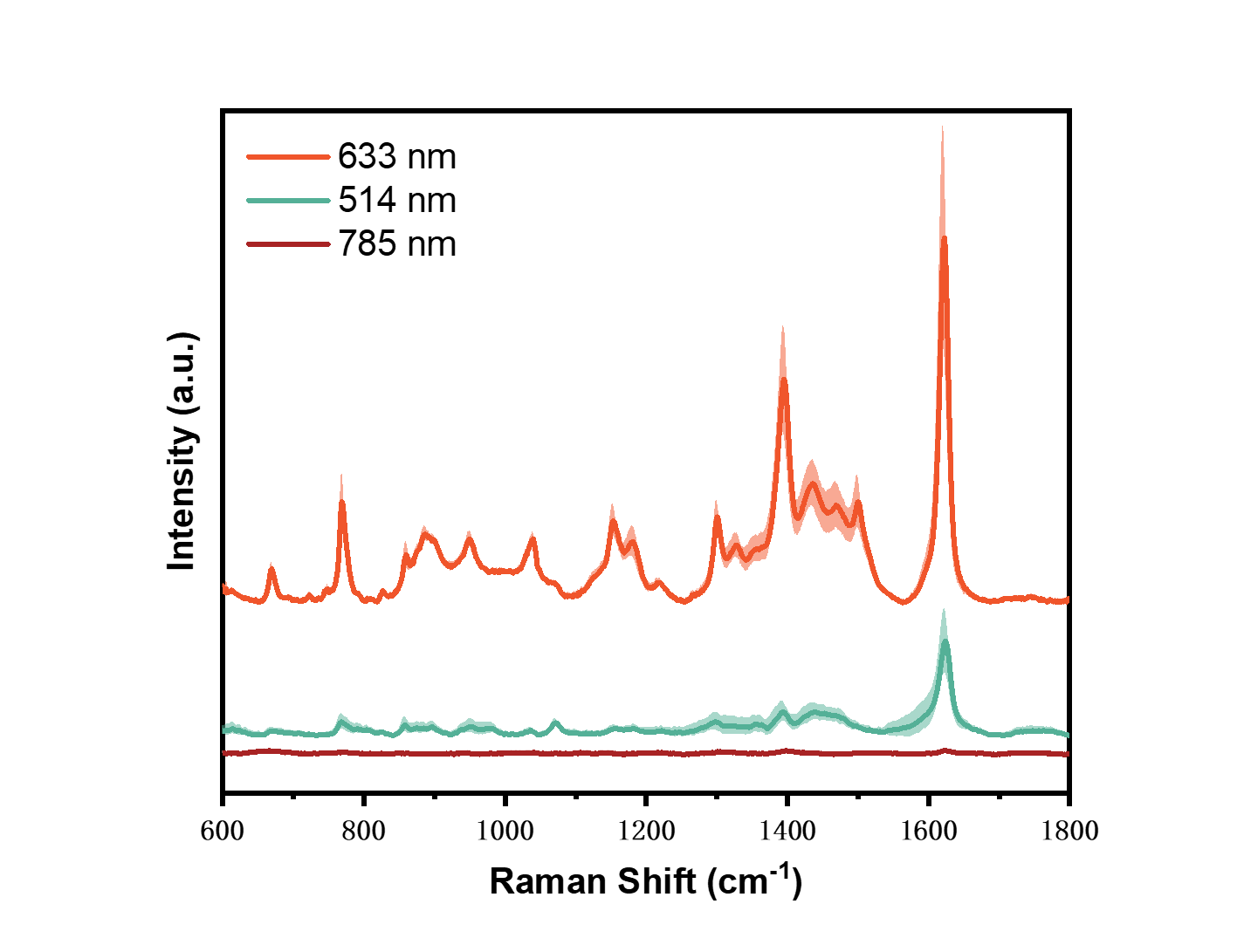


**Figure S11.** Average Raman spectra of 5 × 10^-5^ mol/L methylene blue (MB) absorbed on Cu_1_/UiO-66 under different wavelengths of laser, and the shadow represents the error bars.

**Figure S12.** SERS spectra of MB absorbed on UiO-66 at different concentrations.

**Table S4.** Raman spectral peak assignment of MB and MO

| Peak / cm^-1^ | Assignment |
| --- | --- |
| MB | |
| 1623 | ν(C-C) ring |
| 1502 | ν_asym_(C–C) |
| 1438 | ν_asym_(C–N) |
| 1395 | α(C–H) |
| 1301 | α(C–H) |
| 1155 | β(C–H) |
| 1035 | β(C–H) |
| 669 | γ(C-H) |
| MO | |
| 1594 | υ(C=C) |
| 1419 | υ(N=N) |
| 1392 | υ_α_(C–)SO_2_(–O) |
| 1368 | υ(C–C), δ(Ph–N) |
| 1314 | δ(C–N), υ(C–C) |
| 1195 | *δ*(C–N) |
| 1145 | ν(C-C), ν(C-N) |
| 1117 | υ_s_(C–)SO_2_(–O) |

**Figure S13.** Statistical analysis of the SERS characteristic peak intensity of MB at 1623 cm^-1^.

**Figure S14.** Intensity distribution of the SERS characteristic peak of MB at 1625 cm^-1^.

**Figure S15.** Statistical analysis of the SERS characteristic peak intensity of MO at 1595 cm^-1^.

**Figure S16.** Intensity distribution of the SERS characteristic peak of MO at 1595 cm^-1^.


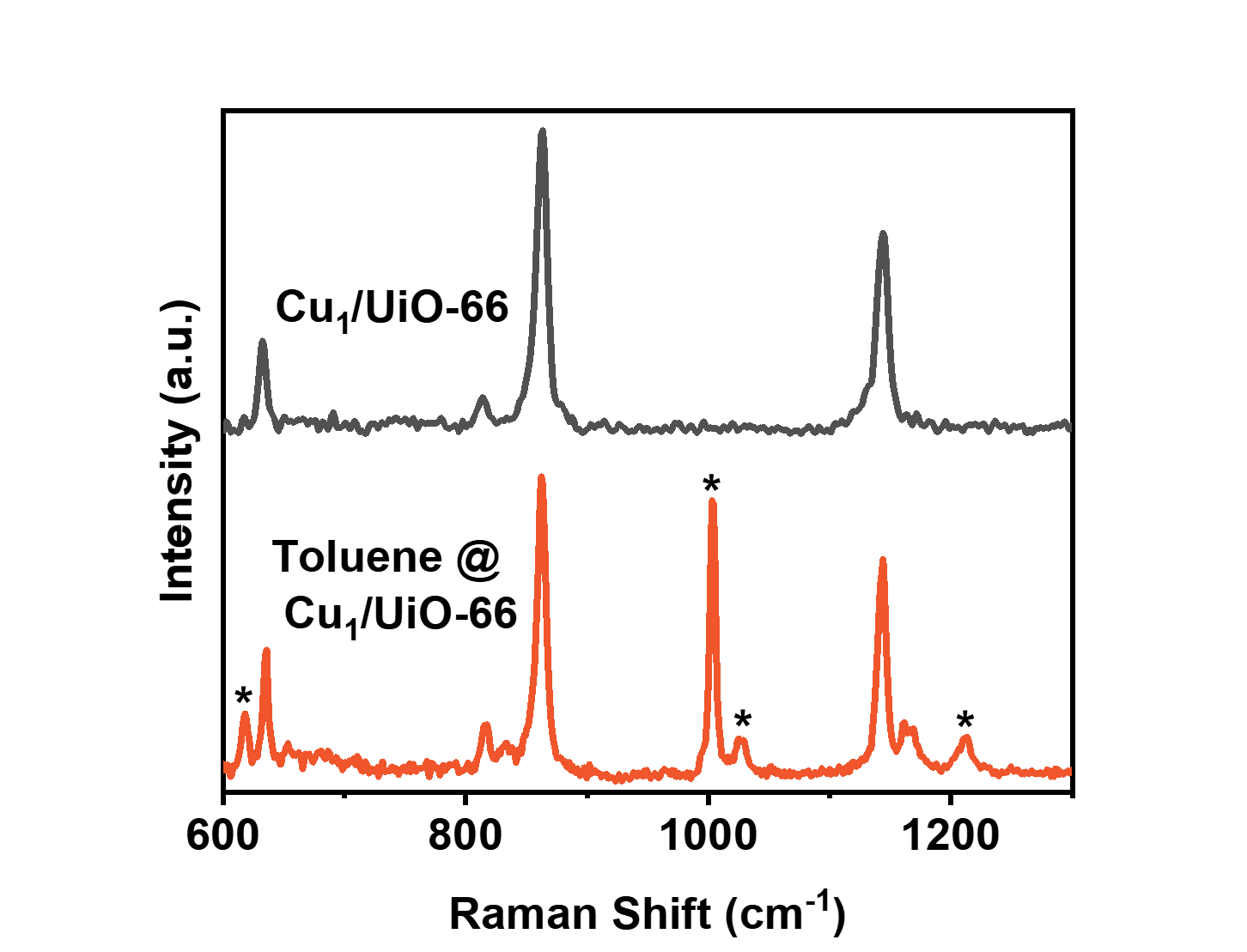


**Figure S17.** Raman spectra of Cu_1_/UiO-66 and toluene (1000 ppm) absorbed on Cu_1_/UiO-66. The characteristic peaks of toluene adsorbed on Cu_1_/UiO-66 are marked with asterisks (*).

**Table S5.** Peak assignment of Cu_1_/UiO-66 and toluene.

| Peak / cm^-1^ | Assignment |
| --- | --- |
| Cu_1_/UiO-66 | |
| 1144 | Terephthalates rings breathing and benzoate rings deformation |
| 863 | Benzene ring breathing |
| 815 | OH bending + CH bending (antiphase) |
| 632 | Benzene ring deformation in terephthalates |
| Toluene | |
| 1210 | C−H bending vibration |
| 1030 | CH_3_—C—CH bending vibration |
| 1003 | stretching vibration of the benzene ring |
| 618 | C=C-C bending vibration |


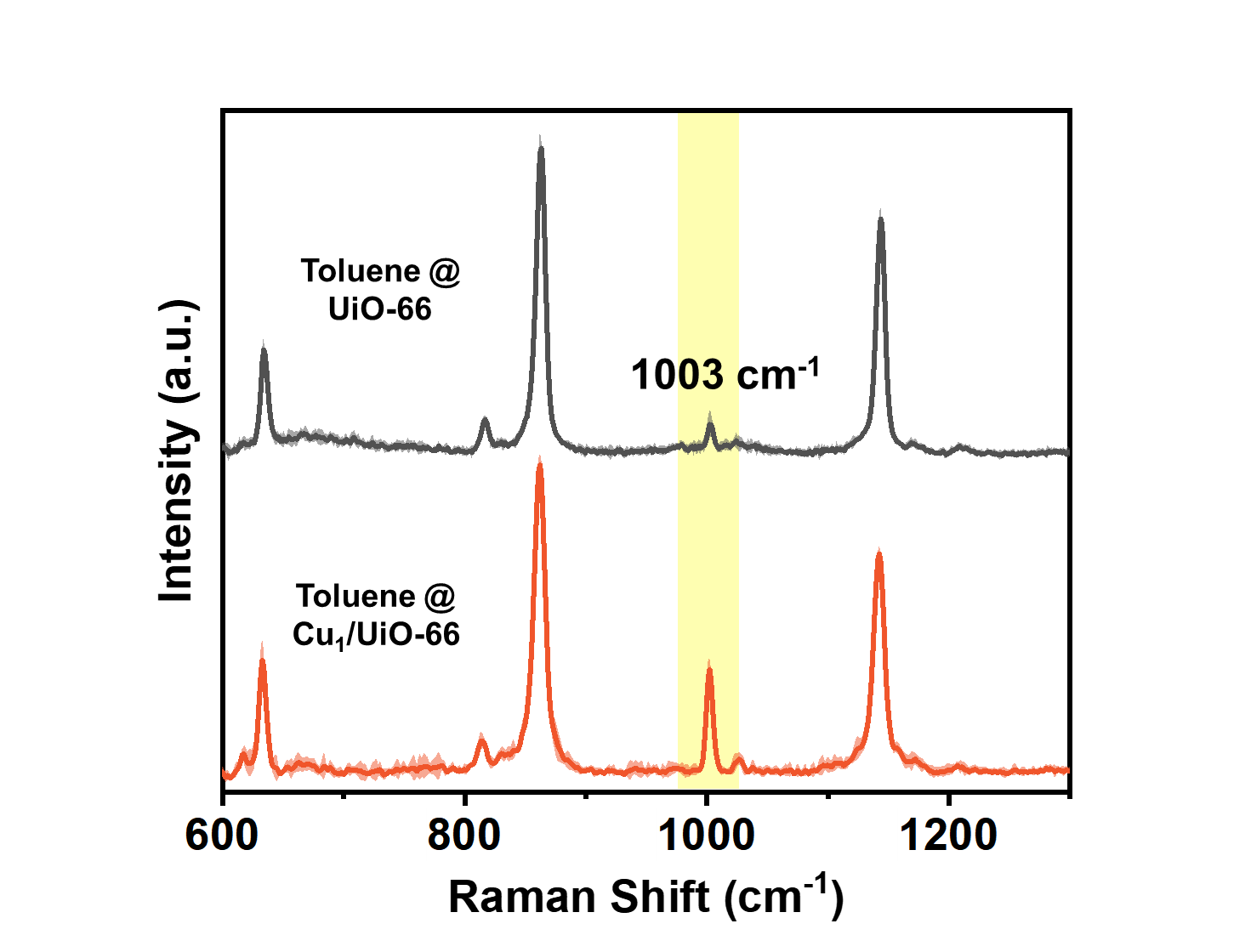


**Figure S18.** Average Raman spectra of toluene (100 ppm) absorbed in UiO-66(black) and Cu_1_/UiO-66 (orange). Each curve was obtained by averaging 10 curves, and the shadow represents the error bars.

**Figure S19.** The SERS spectrum of pure toluene (10 ppm, black) adsorbed on Cu_1_/UiO-66 is compared with that of a toluene solution saturated in water (10 ppm toluene in 20000 ppm water, red). The spectrum is normalized using the intrinsic Raman peak of UiO-66 at 863cm^-1^.

**Figure S20.** SERS intensity – concentration curve of toluene absorbed in the Cu_1_/UiO-66.

The toluene concentration-SERS intensity curve follows an S-shaped function fitting equation：

$$y=\frac{\left( x-v_{rev} \right)g_{max}}{1+exp(\frac{x-v_{half}}{dx})}$$

Where the $v_{half}=0.49\pm0.12$, $dx=-0.55\pm0.14$, $g_{max}=3.68\times{10}^{-4}\pm4.79\times{10}^{-5}$, $v_{rev}=-1104.16\pm166.05$.


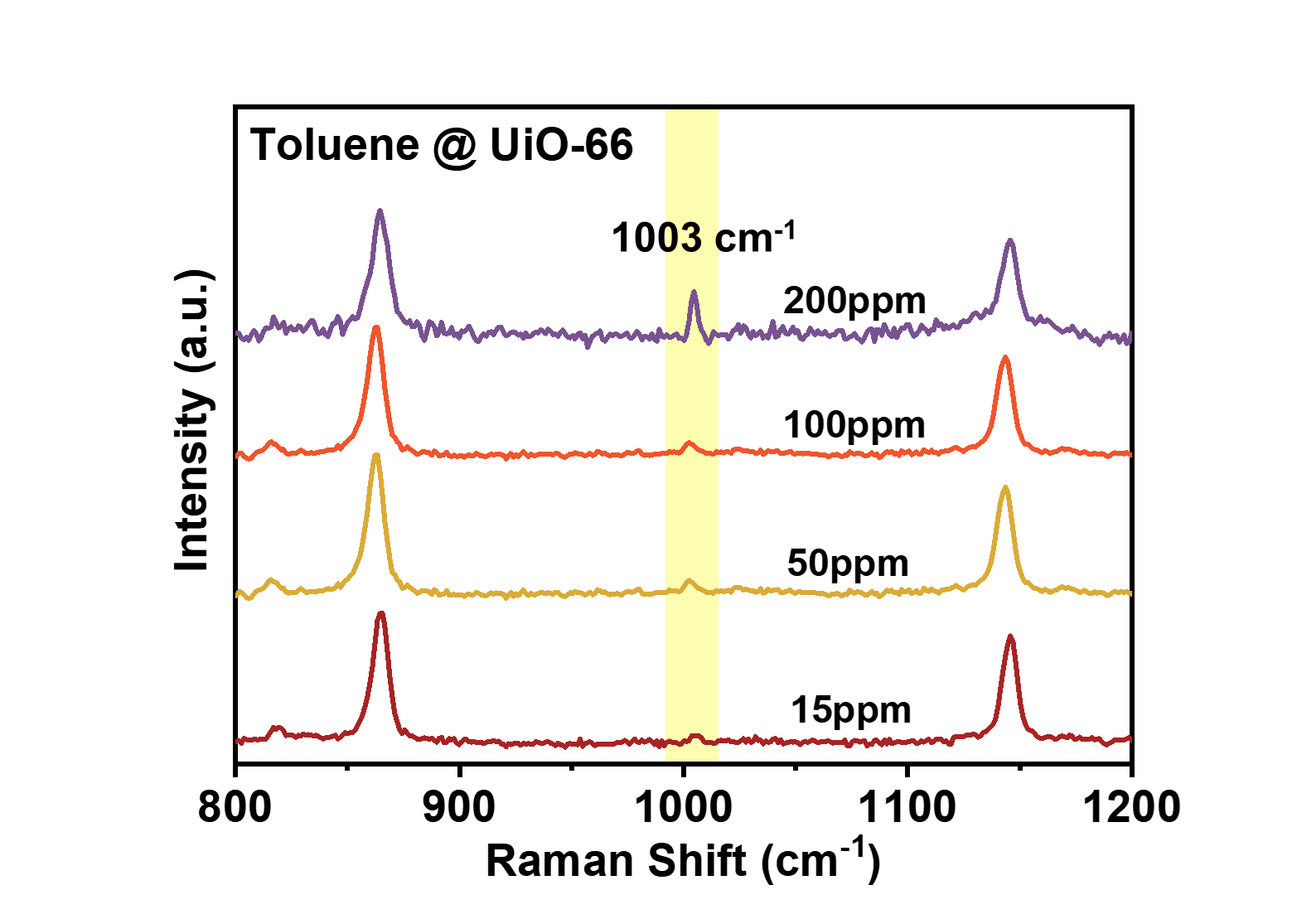


**Figure S21.** Raman spectra of toluene adsorbed on UiO-66 at different concentrations.

**Figure S22.** Time–SERS intensity curve of toluene (100 ppm) absorbed on the Cu_1_/UiO-66.

**Table S6.** Comparison of SERS performance and substrate.

| **Substrate** | **LOD/molecule** | **Ref.** |
| --- | --- | --- |
| AgNPs @ porous 3D silica  **(SPR)** | 68 ppm / toluene | ^1^ |
| AuNPs@UiO-66  **(SPR)** | 1 ppm / toluene | ^2^ |
| PDMS coated Au NPs monolayer film  **(SPR)** | 0.5 ppm / toluene | ^3^ |
| Ag / ZIF  **(SPR)** | 50 ppb / 2-naphthalenethiol | ^4^ |
| Ag/FeCoNi-LDH  **(SPR)** | 10 ppb / benzaldehyde | ^5^ |
| Ti_3_C_2_T_x_ Mxene @ Au nanoarray  **(SPR)** | 10 ppb / benzaldehyde | ^6^ |
| AgNW @ Co-Ni LDH  **(SPR)** | 1.9 ppb / p-ethylbenzaldehyde | ^7^ |
| AgNCs @ Co-Ni LDH  **(SPR)** | 1.83 ppb / benzaldehyde | ^8^ |
| Ni-Fe LDH coated Au nanoarray  **(SPR)** | 1ppb / styrene | ^9^ |
| Ag/silicon membrane  **(SPR)** | 0.5 ppb / anisole | ^10^ |
| Au NPs @ multihole capillaries  **(SPR)** | 0.5 ppb / 4-nitropheol | ^11^ |
| SnO2-NiOx/CuNPs-CuPc  **(SPR)** | 4.1 ppb / 4-ethylbenzaldehyde | ^12^ |
| MIL-100 (Fe) @ AuNPs  **(SPR)** | 0.48 ppb / toluene | ^13^ |
| MIL-100 (Fe)  **(SPR-FREE)** | 2.5 ppm / toluene | ^13^ |
| **Cu_1_/UiO-66**  **(SPR-FREE)** | **10 ppb / toluene** | **THIS WORK** |

**Figure S23.** Statistical analysis of the SERS characteristic peak intensity of toluene at 1003 cm^-1^.

**Figure S24.** Intensity distribution of the SERS characteristic peak of MO at 1003 cm^-1^.

**Figure S25.** Statistical analysis of the SERS characteristic peak intensity of toluene at 1001 cm^-1^.

**Figure S26.** Intensity distribution of the SERS characteristic peak of MO at 1001 cm^-1^.


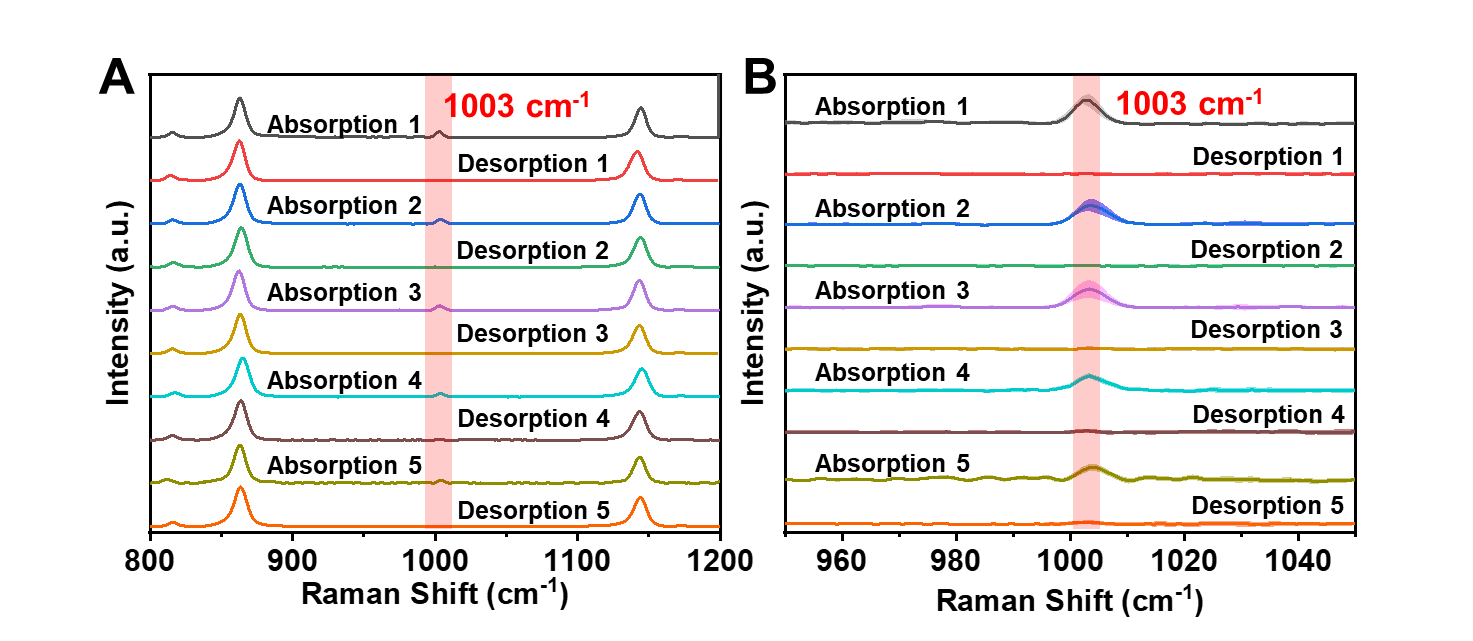


**Figure S27.** (A) Average SERS spectra of Cu₁/UiO-66 obtained from 5 adsorption-desorption cycles, with the toluene characteristic peak at 1003 cm^-1^ highlighted and error bars represented by shaded regions. (B) Zoomed-in view of the average SERS spectra of Cu₁/UiO-66 in the 950-1050 cm^-1^ range, emphasizing the toluene peak at 1003 cm^-1^ with highlighted markers and shaded error bars.

**Figure S28.** SERS intensity at 1003 cm^-1^ during the recycling process.

Characterization and Simulation of the SERS Enhancement Mechanism

**Figure S29.** UV-Vis diffuse reflection spectra of UiO-66 and Cu_1_/UiO-66.


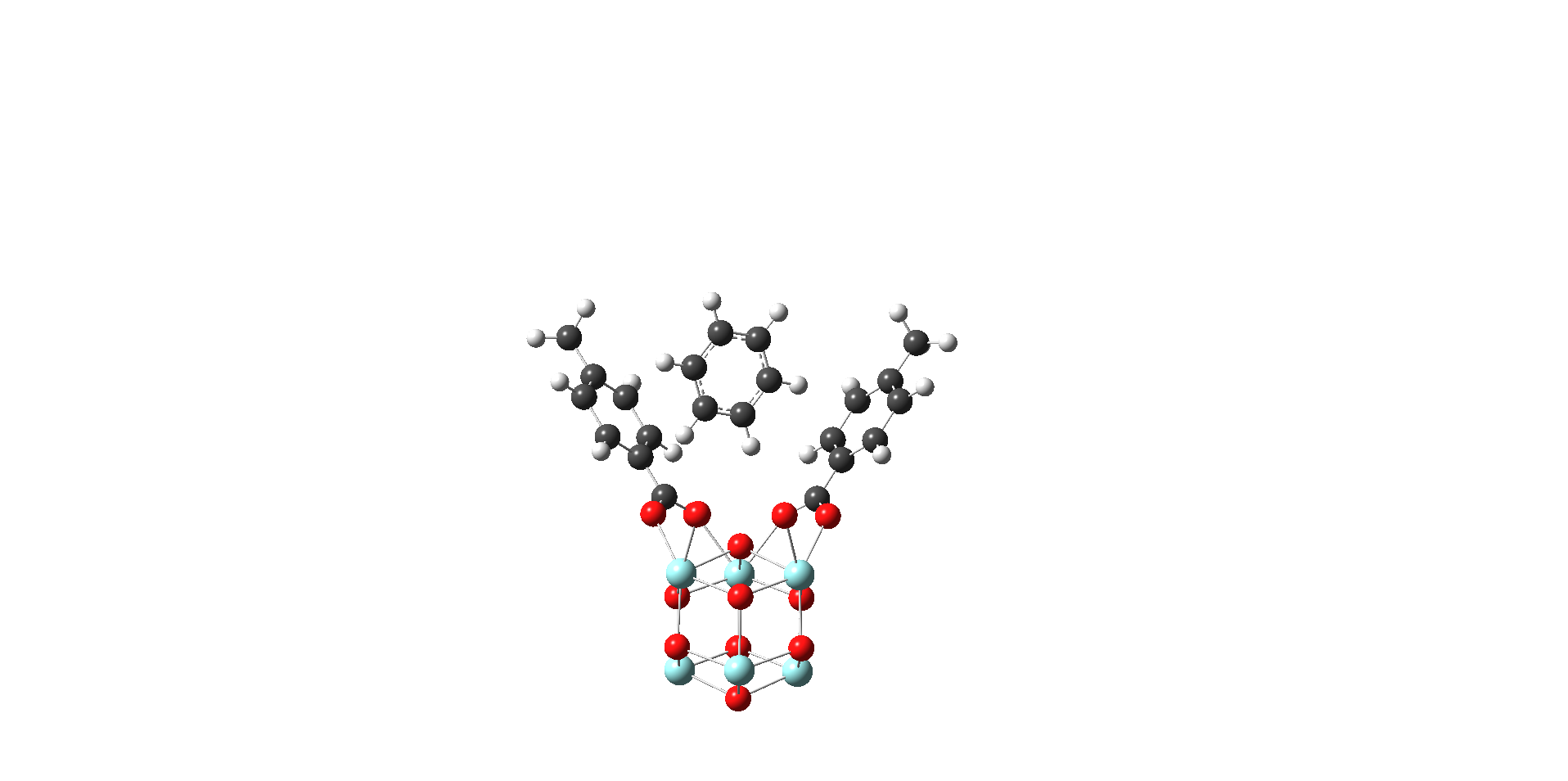


**Figure S30.** Schematic diagram of benzene adsorbed in UiO-66 obtained through DFT calculations.


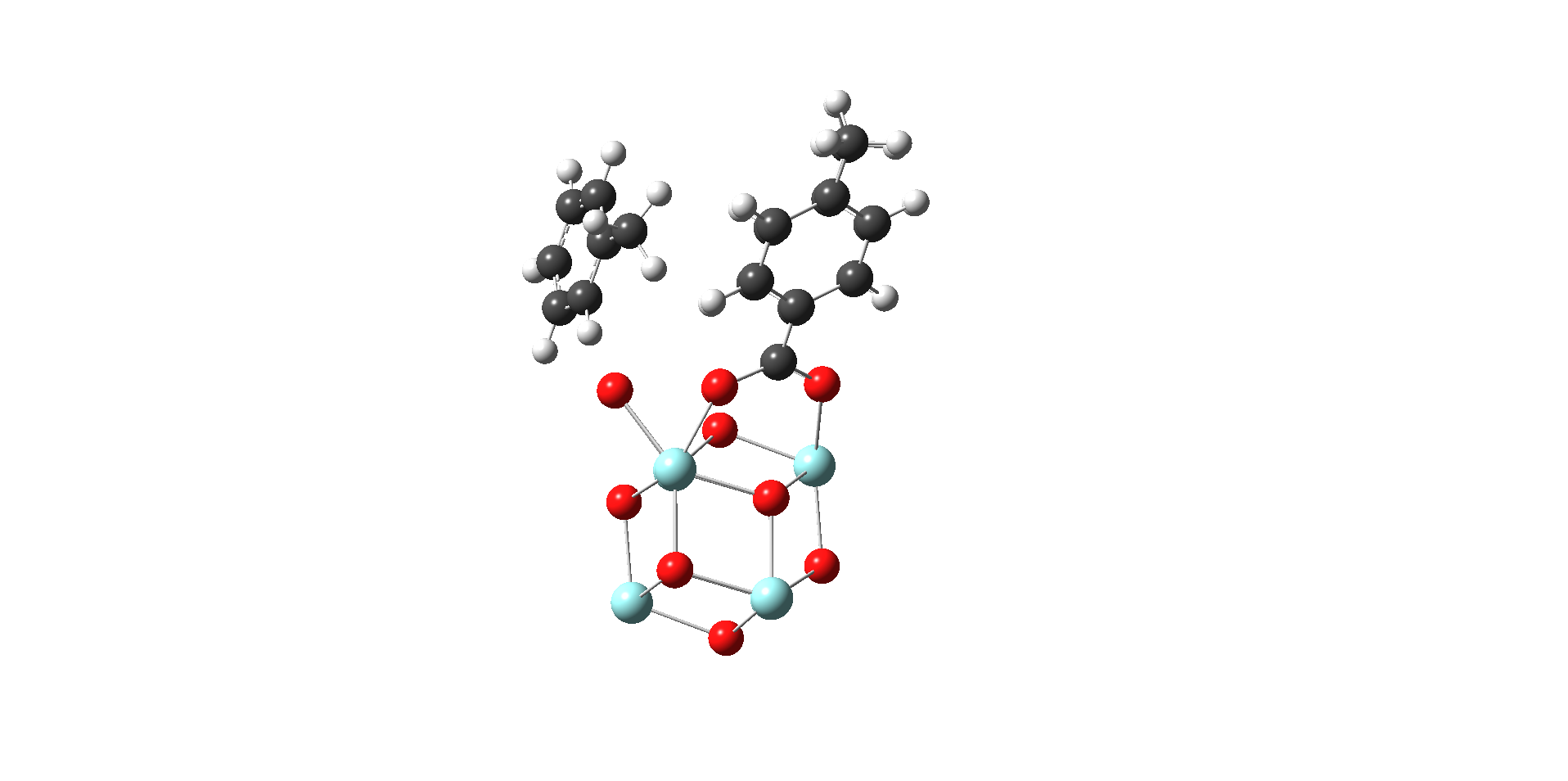


**Figure S31.** Schematic diagram of toluene adsorbed in UiO-66 obtained through DFT calculations.


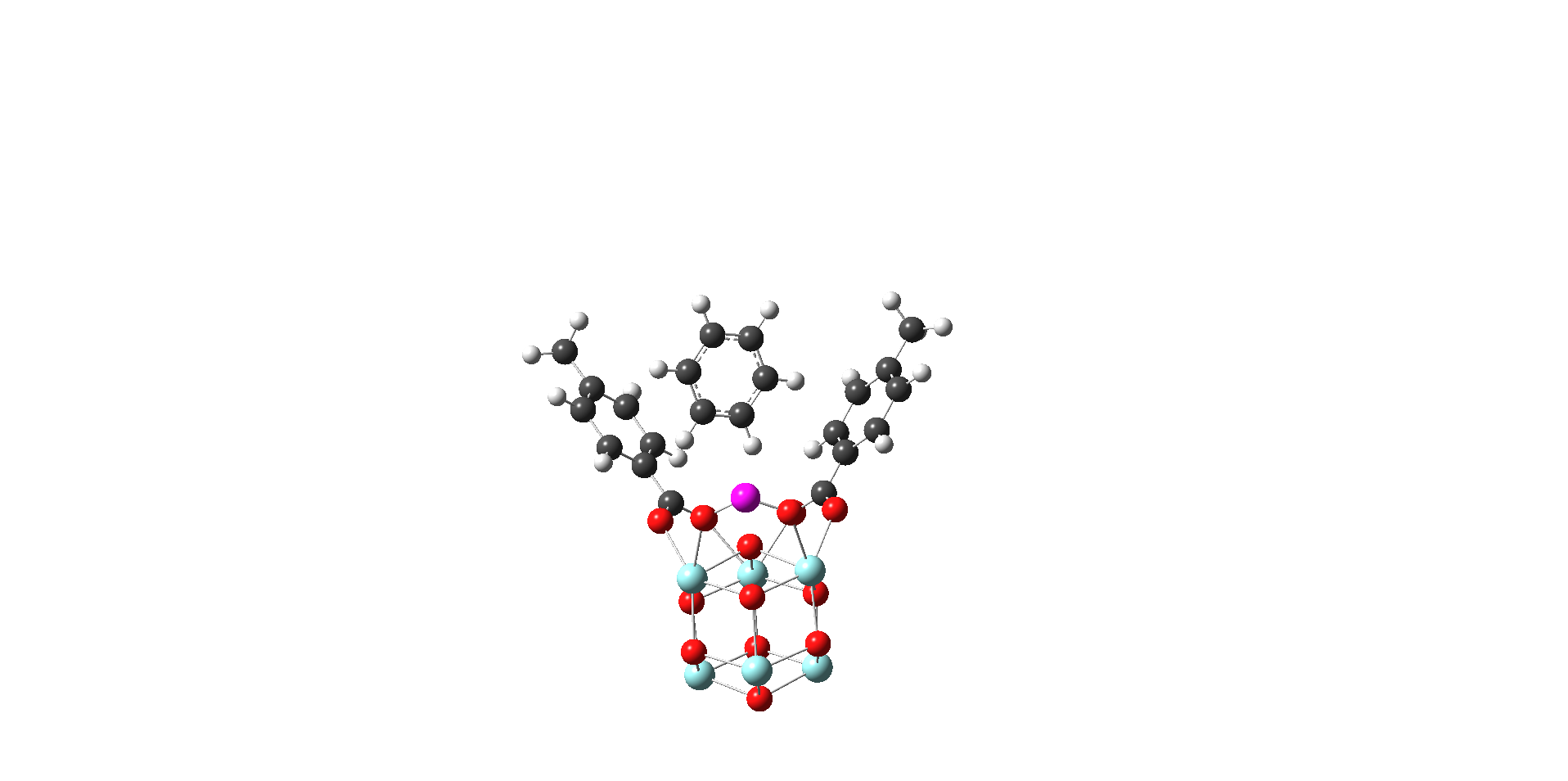


**Figure S32.** Schematic diagram of benzene adsorbed in Cu_1_/UiO-66 obtained through DFT calculations.


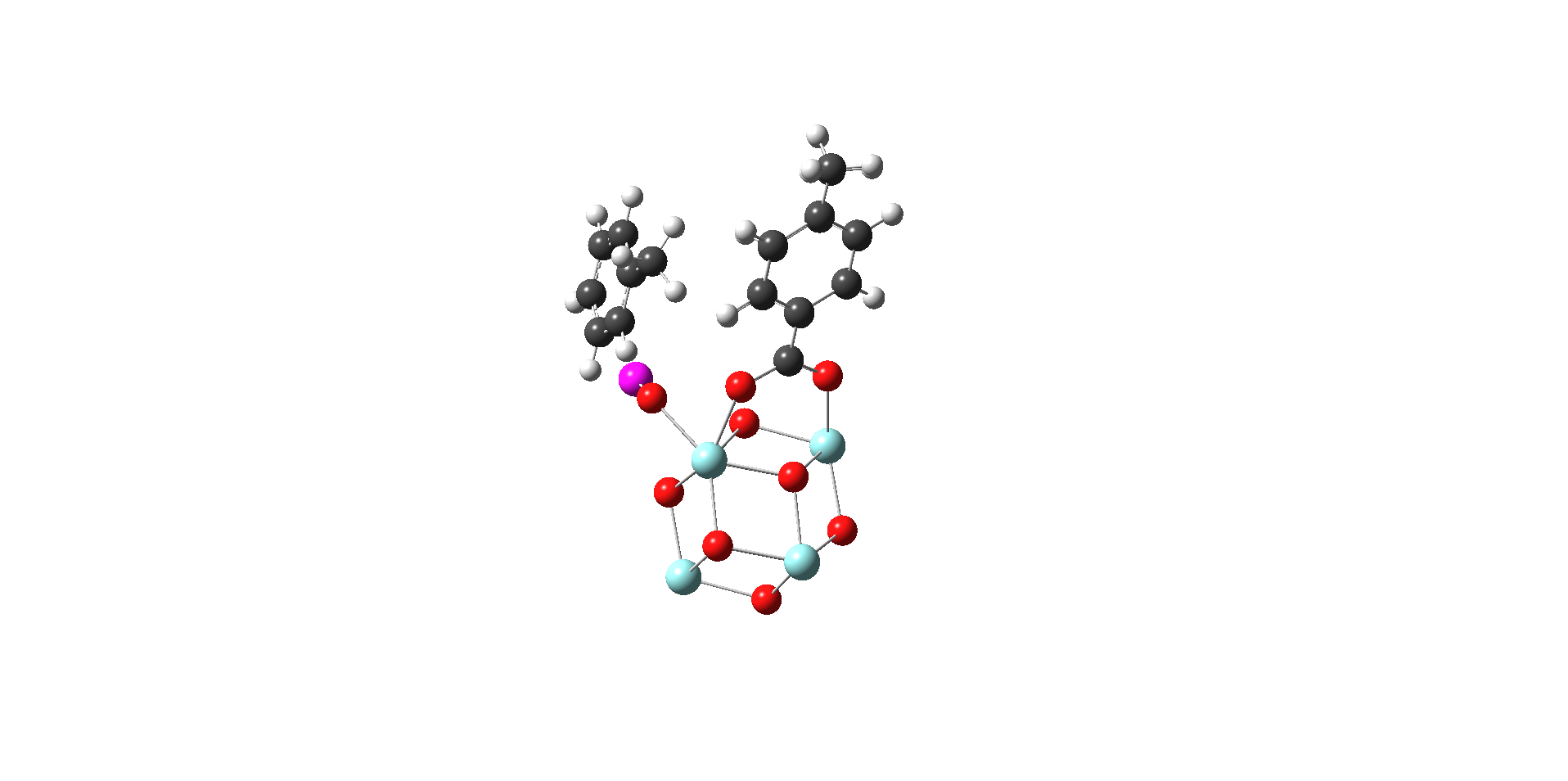


**Figure S33.** Schematic diagram of toluene adsorbed in Cu_1_/UiO-66 obtained through DFT calculations.

Statistical analysis of mixed gas SERS detection

**
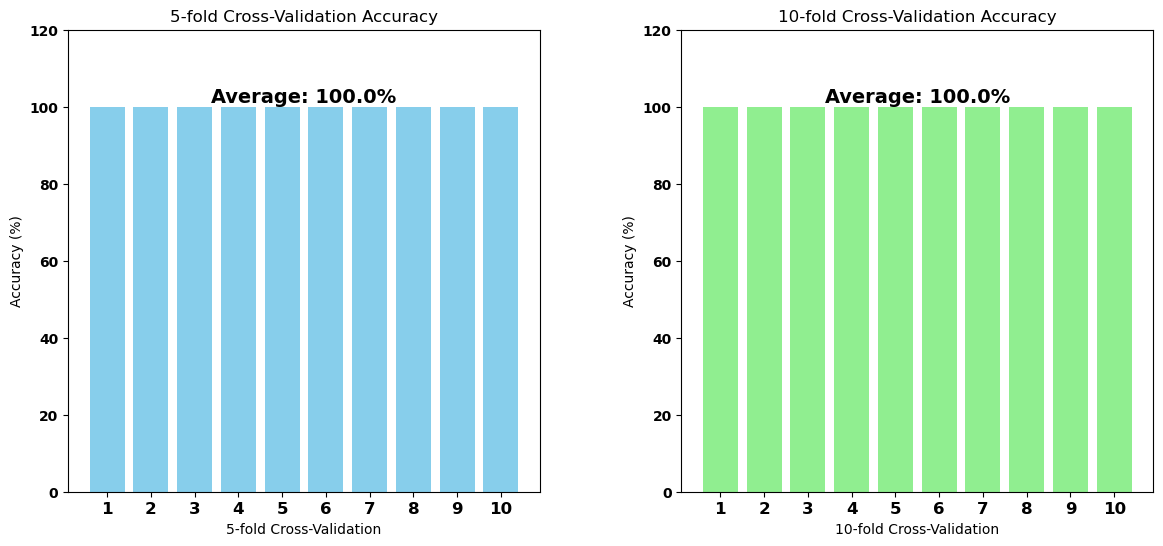
**

**Figure S34.** 5-fold cross-validation and 10-fold validation accuracy of the PCA-LDA method.

**
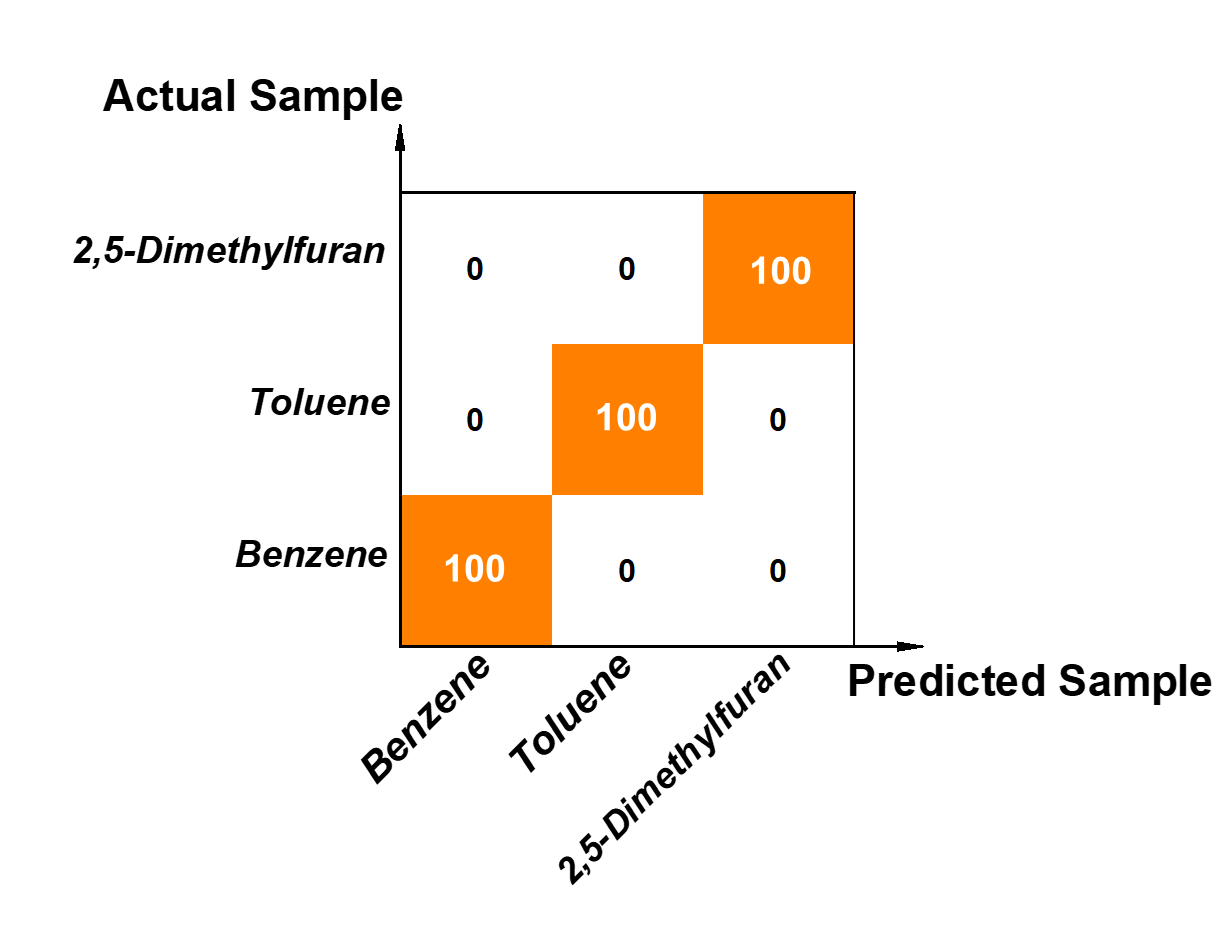
**

**Figure S35.** Confusion matrix of LDA model for 45 test samples. The number is the accuracy (%).
